# Carbendazim shapes microbiome and enhances resistome in the earthworm gut

**DOI:** 10.1186/s40168-022-01261-8

**Published:** 2022-04-18

**Authors:** Jiajin Song, Tongxin Li, Zhiruo Zheng, Wenjie Fu, Zhengnan Long, Nan Shi, Yuling Han, Luqing Zhang, Yunlong Yu, Hua Fang

**Affiliations:** 1 Institute of Pesticide and Environmental Toxicology, College of Agriculture and Biotechnology, Zhejiang University, Hangzhou, 310058 China; 2 Institute of Insect Sciences, College of Agriculture and Biotechnology, Zhejiang University, Hangzhou, 310058 China; 3 Department of Developmental and Cell Biology, University of California, Irvine, CA 92697 USA; 4 Institue of Environmental Health and Pollution Control, School of Environmental Science and Engineering, Guangdong University of Technology, Guangzhou, 510006 China

**Keywords:** Gut microbiota, Fungicide, Manure, Soil animal, Antibiotic resistance genes, Mobile genetic elements

## Abstract

**Background:**

It is worrisome that several pollutants can enhance the abundance of antibiotic resistance genes (ARGs) in the environment, including agricultural fungicides. As an important bioindicator for environmental risk assessment, earthworm is still a neglected focus that the effects of the fungicide carbendazim (CBD) residues on the gut microbiome and resistome are largely unknown. In this study, *Eisenia fetida* was selected to investigate the effects of CBD in the soil-earthworm systems using shotgun metagenomics and qPCR methods.

**Results:**

CBD could significantly perturb bacterial community and enrich specific bacteria mainly belonging to the phylum Actinobacteria. More importantly, CBD could serve as a co-selective agent to elevate the abundance and diversity of ARGs, particularly for some specific types (e.g., multidrug, glycopeptide, tetracycline, and rifamycin resistance genes) in the earthworm gut. Additionally, host tracking analysis suggested that ARGs were mainly carried in some genera of the phyla Actinobacteria and Proteobacteria. Meanwhile, the level of ARGs was positively relevant to the abundance of mobile genetic elements (MGEs) and some representative co-occurrence patterns of ARGs and MGEs (e.g., *cmx*-transposase and *sul1*-integrase) were further found on the metagenome-assembled contigs in the CBD treatments.

**Conclusions:**

It can be concluded that the enhancement effect of CBD on the resistome in the earthworm gut may be attributed to its stress on the gut microbiome and facilitation on the ARGs dissemination mediated by MGEs, which may provide a novel insight into the neglected ecotoxicological risk of the widely used agrochemicals on the gut resistome of earthworm dwelling in soil.

**Video abstract**

**Graphical Abstract:**

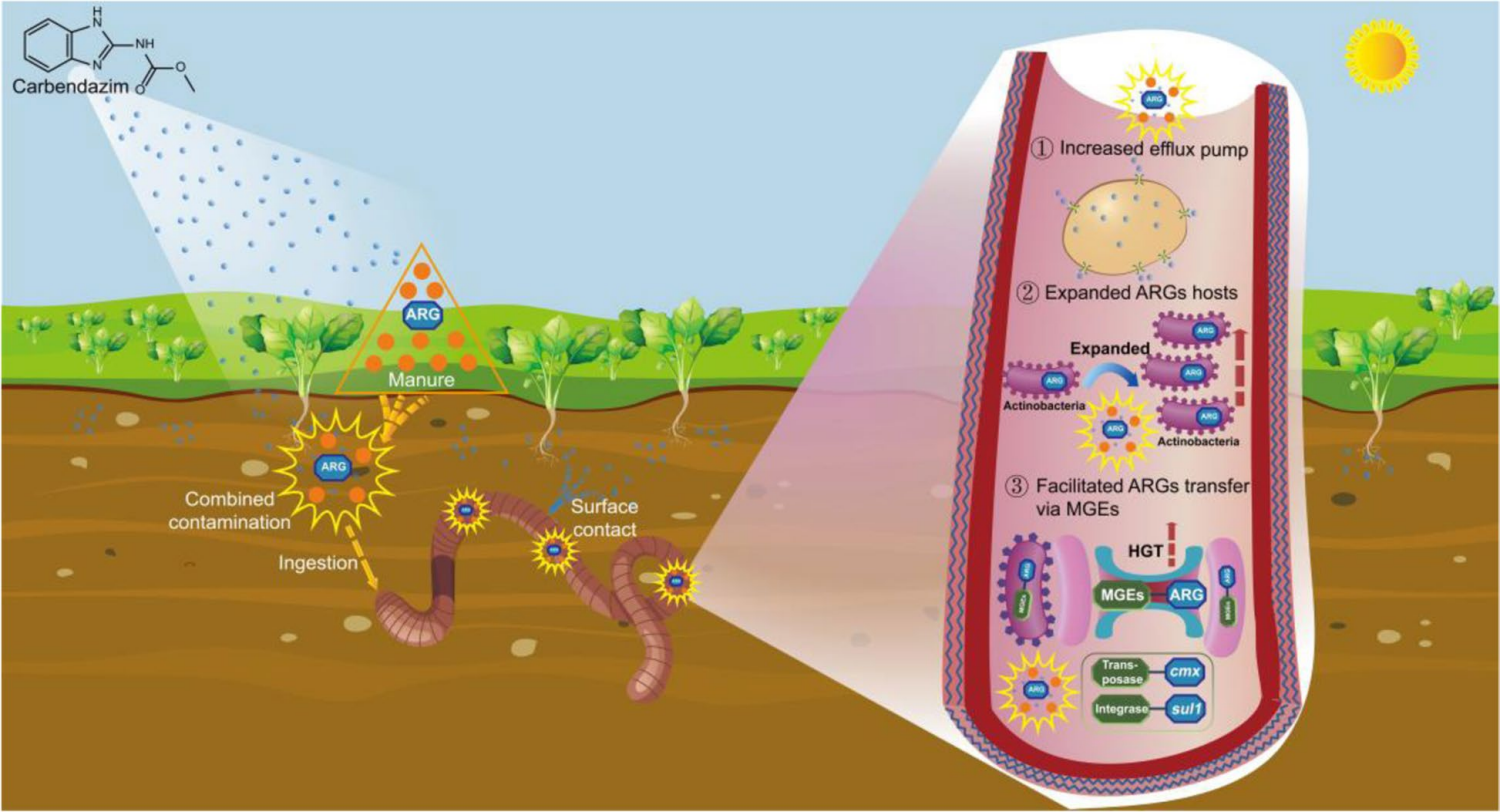

**Supplementary Information:**

The online version contains supplementary material available at 10.1186/s40168-022-01261-8.

## Background

Repeated fertilization with livestock manure in the cultivation of the crops is a vital way for yield enhancement as well as soil quality [1]. However, manure is recognized as a reservoir of antibiotic resistance genes (ARGs) that its fertilization may lead to the emergence and dissemination of ARGs from feedlot to agricultural soil [2, 3]. Meanwhile, fungicide is frequently applied in agricultural production to control fungal diseases, a large proportion of which eventually enter the soil environment leading to fungicide residue contamination [4]. Carbendazim (methyl benzimidazol-2-ylcarbamate, CBD) is a widely used fungicide that contains a benzimidazolic ring leading to its relative long-term retention and residue contamination which was frequently detected at the concentration of μg kg^−1^ to mg kg^−1^ in agricultural soils [5].

Previous studies have reported that CBD residues pose detrimental effects on the growth of soil fauna and enzymatic activity, respiration activity, and community structure of soil microbiota [6–8]. As a typical animal in soil, earthworm plays an important role in soil nutrient cycling, and numerous studies reported that gut microbiota is essential to perform functions for host [9, 10]. Due to the ingestion behavior and surface contact of earthworm, its body tissue and gut may bioaccumulate a considerable amount of CBD residues [11]. Recently, various ARGs have been detected in the gut of soil fauna, and some pollutants (antibiotics, heavy metals, and nonantibiotic carbamazepine, etc.) could exert selective pressures on resistome [12–14]. However, the response of microbiome and resistome to the agricultural fungicide CBD exposure in the earthworm gut from the manured soil remains unclear.

In this study, the representative soil fauna earthworm (*Eisenia fetida*) was selected to investigate the effects of CBD on the gut microbiome and resistome in the manured soil-earthworm ecosystem using shotgun metagenomics and quantitative polymerase chain reaction (qPCR) methods. The aims of this study were (1) to measure dissipation and bioaccumulation characteristics of CBD in the earthworm and soil, (2) to reveal the response of gut microbiome and resistome to CBD exposure, (3) to explore the relationship between ARGs and microbial community in the earthworm gut, and (4) to analyze the co-occurrence patterns between ARGs and MGEs in the earthworm gut under CBD exposure. Overall, the results of this study would strongly broaden the current knowledge about the role of agrochemicals in gut resistome of soil fauna and provide a novel insight into the potential ecological risk of fungicides.

## Materials and methods

### Chemical, soil, and earthworm

Technical grade fungicide CBD (purity ≥ 98%) was purchased from Aoke Biology Research Co. The soil was collected at the depth of 0~15 cm from a mulberry field in the Huajiachi Campus of Zhejiang University, Hangzhou, China, which had no history of fungicide or manure application. After air dried, the collected soil samples were sieved (2 mm) and all stones and debris were removed. The chicken manure was purchased from a farm in Jiaxing, China, and the detailed physiochemical properties of the collected soil and manure are summarized in Table S1. Manure (3%, w/w) was mixed into the soil to simulate the manure-amended soil (MS) in agricultural production, while no manure amended soil (NS) was used as the control. All soil samples were pre-incubated in an artificial climate room (25 °C) for several days to reach a balanced and stable state. The earthworm (*E. fetida*) was purchased from a farm in Jiangsu, China, and then cultured for more than one month under laboratory conditions. Before the pot experiment, sexually mature earthworms with similar biomass were selected and transferred to a glass beaker of which the bottom was laid two pieces of filter paper with some sterile water for 24-h starvation treatment in darkness to ensure that the gut content was empty.

### Pot experiment and sample collection

The mother solution of CBD was prepared using N, N-dimethylformamide (DMF), and gradually diluted with water to a series of standard solutions of CBD. CBD standard solution was sprayed to the 400 g of soils and then mixed completely to achieve two final concentrations of 1.0 mg kg^−1^ (CBD1) and 2.0 mg kg^−1^ (CBD2), while the soils without CBD were set as the control. The concentrations of CBD in the soil were set by comprehensively considering the recommended dosage, the actual environmental residue level, and the toxicity to *E. fetida* in soil [7]. The soil water content was adjusted to 60% of the maximum water holding capacity with sterile deionized water. Subsequently, all soils were transferred into the plastic pots (upper diameter 150 mm, height 85 mm, lower diameter 103 mm). Twenty *E. fetida* individuals were placed onto the soils in each pot that were covered with aluminum foil with several 1 mm holes. All pots were incubated for 28 days at 20 ± 1 °C, with a 75% relative humidity and a 12:12-h dark/light photoperiod in an artificial climatic chamber. The pots were weighed every 2 days and the water loss was supplemented by adding an equal volume of sterile deionized water to maintain the water content during the incubation period [15]. All treatments were conducted in triplicate. At the 0, 1, 3, 7, 14, 21, and 28 days after exposure to CBD, 20.0 g of soil samples and several earthworms (none at 0 or 1 days) were randomly collected for the determination of CBD residues and total DNA extraction. The collected samples were marked according to origin of samples (G-gut and S-soil).

### Determination of CBD residues in soil and earthworm

CBD residues in the soil were determined according to the modified methods [16]. Before CBD extraction from the earthworms, the gut content of earthworms was empty overnight. About 2.0 g of earthworms or soils were crushed by a tissue crusher in the presence of 8 ml of acetonitrile-water (1:1) solution and oscillated for 10 min. Afterward, the mixture was ultrasonically extracted for 30 min, oscillated for 5 min by adding 1.0 g of NaCl and 2.0 g of anhydrous MgSO_4_, and then centrifugated for 5 min. Subsequently, 100 mg of PSA and 50 mg of C18 were added and oscillated for 1 min and centrifugated for 5 min. Finally, the supernatant was filtered through a 0.22-μm organic filter membrane and detected by HPLC.

### Dissection of earthworm gut

The collected earthworms were rinsed for five times in sterile water. Subsequently, the earthworms were placed in plastic containers upon ice for 10 min to prevent casting, immersed in 75% ethanol for sterilization purposes and then washed for five times with sterile water. To obtain the earthworm gut, dissection was operated. In brief, the body tissues surrounding the gut were cut open using sterile scissors, dissecting needles, and forceps under sterile conditions. The body tissues were discarded to reduce the host contamination. The gut portions behind the gizzard were collected as the gut samples (approximately 1.0 g) into a 2-ml tube containing 1 ml of phosphate buffer solution (0.1 mol/L and pH = 7.0), and then mixed for 1 min in a vortex mixer. The impurities containing earthworm coelomic fluid in the earthworm gut content were discarded by washing thrice with the same phosphate buffer solution as above. The obtained gut samples were stored at − 80 °C for subsequent DNA extraction.

### DNA extraction and metagenomic sequencing

Total DNA from the soil and earthworm gut samples were extracted using FastDNA SPIN for Soil Kit (MPBio Laboratories, USA) following the manufacturers’ protocol in triplicate. The extracted DNA concentration and purity were measured using NanoDrop 1000 (Thermo Fisher Scientific, Waltham, MA, USA). The sequencing libraries (250 bp fragment) were prepared according to the manufacturers’ recommendations and sequenced on an Illumina NovaSeq 6000 platform at Novogene (Tianjin, China). All raw data has been deposited in Sequence Read Archive (NCBI) under the BioProject of PRJNA773059. The raw reads with low quality (< 20) and ambiguous nucleotides (*>* 3) were trimmed using the fastp software at default settings to guarantee the quality of downstream metagenomic analysis [17]. For the reads from the gut samples, Bowtie2 was applied to remove the host contamination using the reference genomes of *E. fetida* (GenBank: GCA_003999395.1 and GCA_900000155.1) by the “very-sensitive” mode [18]. The information of the metagenomic dataset in each sample is listed in Table S2.

### Microbiome analysis

The analysis of microbiome in the earthworm gut and soil was performed using Kraken2 and Bracken software based on the clean reads of metagenomics [19, 20]. Briefly, Kraken2, together with a customized complete genome k-mer database, was applied to clean reads [20]. The classification results were further passed through Bracken for relative abundance estimation of the taxa in each sample [19].

### Characterization and quantification of ARGs/MGEs

For the ARGs characterization, the clean reads were searched against the modified homology protein sequences (the global regulatory proteins and mutants were removed) of the Comprehensive Antibiotic Resistance Database (CARD, Aug 2020) using BLASTX algorithm with an *e* value cutoff of 1e−5 [21]. The best hit results were filtered with an identity cutoff of 80% and an alignment length cutoff of 25 amino acids [3], and the remaining hit was annotated as ARG-like sequence. The resistance types (e.g., tetracycline and sulfonamides), subtypes (e.g., *tetW* and *sul1*), and resistance mechanisms were classified using a customized script [15]. Meanwhile, the pair-end reads were searched against a simplified MGEs database using the Bowtie2 with the parameters mentioned in a preceding study to identify MGEs [22]. To assess the level of ARGs and MGEs, the abundance of these genes was normalized to the size of bacterial communities (i.e., copy of 16S rRNA gene) using the previous method [23]. To validate the absolute abundance of ARGs in these samples, ten abundant ARGs including seven multidrug resistance genes (i.e., *ceoB*, *acrB*, *mexF*, *mexK*, *muxB*, *mtrA*, and *mdtB*), two peptide resistance genes (i.e., *vanRO* and *vanSO*), and a sulfonamides resistance gene (i.e., *sul1*) were selected for quantification using qPCR performed on Applied Biosystems QuantStudio3 (ThermoFisher Scientific, USA) [24], and the corresponding primers are listed in Table S3.

### Metagenomic assembly, gene prediction, and functional annotation

Metagenomic assembly of short reads into contigs was performed using metaSPAdes software with a default k-mer size list [25]. 165,731 to 316,773 contigs were obtained for every treatment. The open reading frame (ORF) prediction of the contigs (≥ 500 bp) was performed by Prodigal with the parameter “-p meta” [26]. ARG-like ORFs were determined using BLASTP against the CARD at an e-value cutoff of 1e−5 with a minimum identity of 80% and a lowest query sequence coverage of 70%. According to the previous study [27], the amino acid sequences of ORFs were also used to match against the non-redundant protein database for MGEs identification using the DIAMOND program at the same setting [28]. The co-occurrence arrangements of ARGs and MGEs were picked out if they were simultaneously located on the identical contigs [29]. Moreover, the contigs containing ARG-like ORFs were defined as AR-contigs (ARCs). ARCs were matched against the non-redundant protein database for bacteria by Kaiju software to track the hosts of ARGs [30] and classified (chromosome- or plasmid-origin) by PlasClass [31].

### Statistical analysis and visualization

Statistical comparisons of resistome and bacterial taxa were analyzed using *t* test and one-way analysis of variance (ANOVA) with a post hoc Tukey HSD test or Kruskal-Wallis test, depending on the results from Levene’s test of homogeneity of variances between the treatments. Analysis of differences (ANOSIM) of ARGs profiles and bacterial communities was performed between treatments in soil and gut samples. The principal coordinates analysis (PCoA) was conducted based on the Bray-Curtis distances using the “vegan” package of R. Pearson’s correlation analysis was performed to explore the internal relationships between the abundance of ARGs and MGEs with the “corrplot” package. The bipartite network was used to uncover the unique and shared subtypes of ARGs between different treatments and the tracking network was conducted to show the potential hosts of ARGs, both of which were constructed using “vegan”, “hmisc”, and “igraph” packages in the R and Gephi software for visualization purposes [32].

## Results

### Dissipation and bioaccumulation of CBD in the soil-earthworm systems

**Fig. 1:**
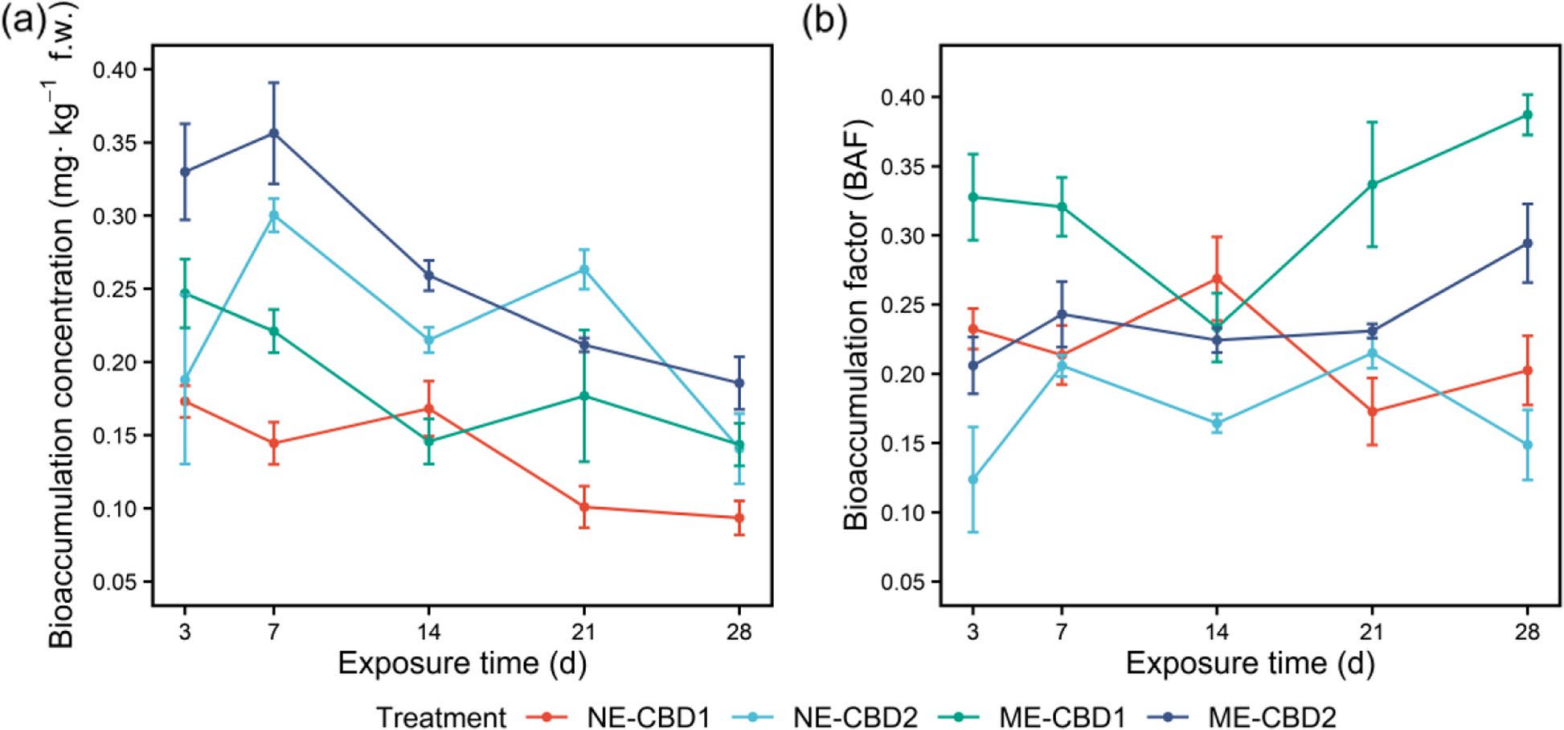
Bioaccumulation concentration (**a**) and bioaccumulation factor (BAF, **b**) of carbendazim in the earthworm among treatments. NE-CBD1 and NE-CBD2 represent the earthworm samples in the un-manured soil with 1.0 and 2.0 mg kg^−1^ CBD, respectively. ME-CBD1 and ME-CBD2 represent the earthworm samples in the manured soil with 1.0 and 2.0 mg kg^−1^ CBD, respectively

In the soil-earthworm systems*,* earthworms were exposed to different doses of CBD in the soil for 4 weeks. As shown in Fig. 1a, the bioaccumulation concentration of CBD in the earthworm decreased gradually with the extension of treatment time, and the final CBD residual levels were 0.09 (NG-CBD1), 0.14 (MG-CBD1), 0.14 (NG-CBD2), and 0.19 (MG-CBD2) mg kg^−1^ f.w., respectively. The addition of manure in the soil enhanced the bioaccumulation factors (BAF) of CBD in the earthworm (Fig. 1b). Meanwhile, the dissipation of CBD in soils followed the first-order kinetics equation (0.9402 < *r <* 0.9936) and the half-lives were 33.00 days (NS-CBD1), 23.49 days (MS-CBD1), 33.16 days (NS-CBD2), and 19.15 days (MS-CBD2), respectively (Table S4). The final CBD residual levels were detected to be 0.46 mg kg^−1^ in the NS-CBD1, 0.37 mg kg^−1^ in the MS-CBD1, 0.947 mg kg^−1^ in the NS-CBD2, 0.63 mg kg^−1^ in the MS-CBD2, respectively, after 28 days of exposure (Figure S1). The biomass of earthworm increased at the end of 28 days cultivation but was not significantly (one-way ANOVA, *p >* 0.05) different in the CBD treatments as well as the survival rate (Figure S2 and Table S5), implying that the treatment concentrations of CBD in the soil had no obvious acute toxicity to the earthworms.

### Effects of CBD on the gut microbiome

**Fig. 2:**
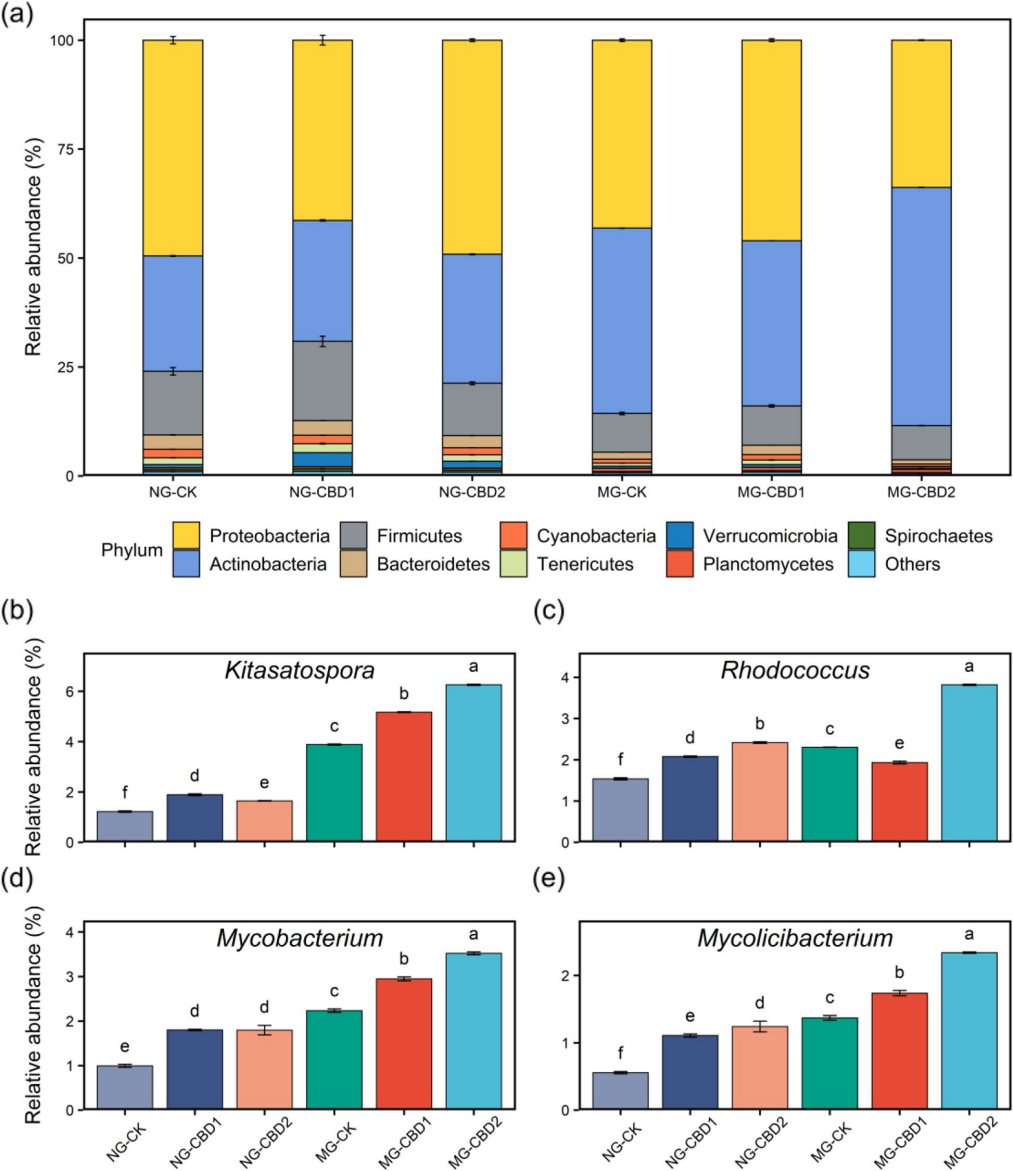
Composition of microbiota at phylum level (**a**) and relative abundance of the genera belonging to Actinobacteria (**b**) with significant differences in the earthworm gut among treatments. NG-CK, NG-CBD1, and NG-CBD2 represent the earthworm gut samples in the un-manured soil with 0, 1.0, and 2.0 mg kg^−1^ CBD, respectively. MG-CK, MG-CBD1, and MG-CBD2 represent the earthworm gut samples in the manured soil with 0, 1.0, and 2.0 mg kg^−1^ CBD, respectively

As shown in Fig. 2a, Proteobacteria (33.9–49.4%), Actinobacteria (26.5–54.6%), Firmicutes (7.86–18.2%), and Bacteroidetes (0.972–3.38%) were the dominant phyla, which together accounted for more than 90% of the gut microbiota. CBD exposure altered the composition of the gut microbiome at the phylum level. Compared to the control, the relative abundance of Actinobacteria significantly (*p <* 0.05) increased by 111.7% in the NG-CBD2 and 128.4% in the MG-CBD2 while Proteobacteria (*p <* 0.05) notably decreased by 21.6% in the MG-CBD2. However, the relative abundance of some phyla (e.g., Firmicutes and Planctomycetes) did not remarkably (*p >* 0.05) change under CBD exposure. Concerning the alpha diversity, the Shannon diversity index fluctuated from 1.10 to 1.54 in the gut and displayed a stimulation-recovery-suppression trend during the CBD exposure (Figure S3a). The heatmap of the dominant genera (top 50) uncovered the diverse responses of the gut and soil microbiota under the CBD exposure (Figure S4). The dominant bacterial genera were *Burkholderia*, *Streptomyces*, *Microbactertium*, *Bacillus*, and *Achromobacter* in the earthworm gut, and *Paraburkholderia*, *Cupriavidus*, *Streptomyces*, *Burkholderia*, and *Pseudomonas* in the soil. However, a significant correlation between the microbiota of soil and earthworm gut was found using the Mantel test (*r* = 0.79, *p <* 0.0001). Noteworthily, the relative abundance of *Burkholderia*, *Bradyrhizobium*, and *Klebsiella* (Proteobacteria) dramatically declined (*p <* 0.05) while *Streptomyces* (Actinobacteria) significantly increased (*p <* 0.05) in the MG-CBD2 (Figure S5). Whereas, the relative abundance of some genera belonging to the phylum Actinobacteria, such as *Kitasatospora*, *Rhodococcus*, *Mycobacterium*, and *Mycolicibacterium*, significantly increased in the earthworm gut with the increasing CBD concentrations (Fig. 2b). As shown in Figure S6a, the PCoA based on the Bray-Curtis dissimilarity showed a clear separation pattern in the gut bacterial communities at the genus level between treatments along the PCoA 1-axis (*p <* 0.05; ANOSIM), which was also found in the soil (Figure S6b).

### Effects of CBD on the gut resistome

**Fig. 3:**
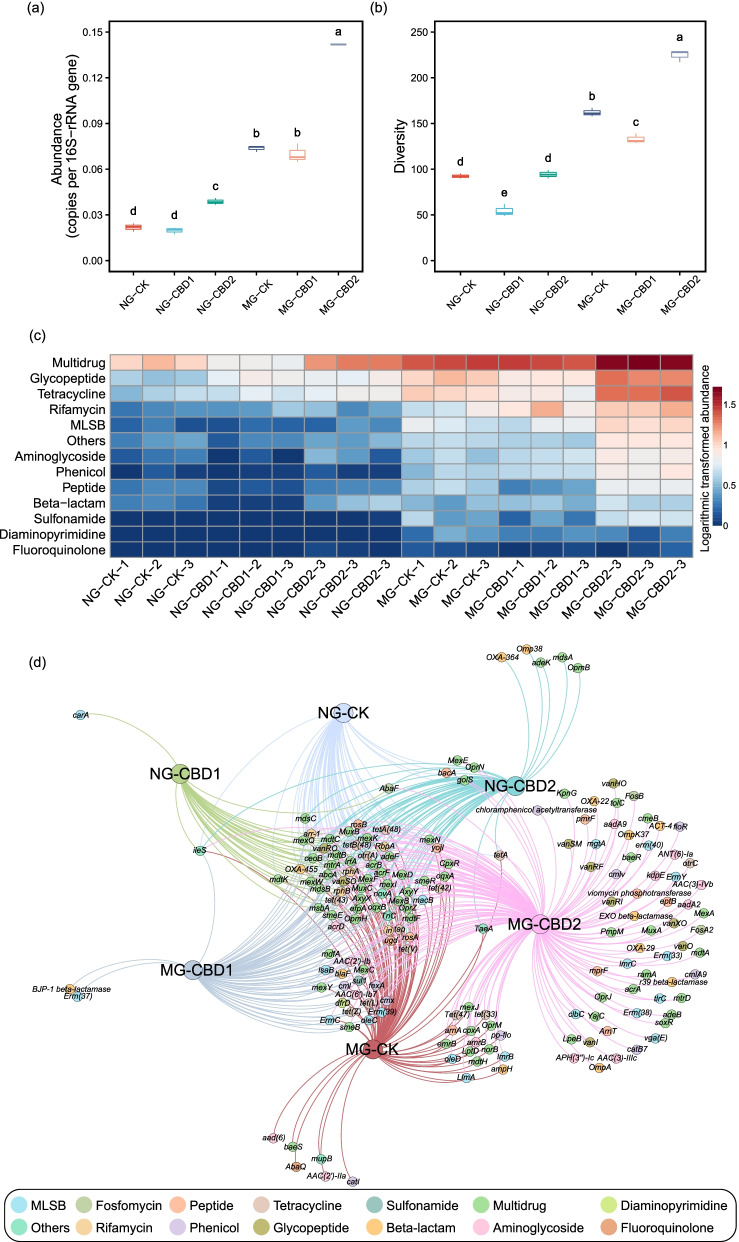
Comparison of total abundance (**a**) and diversity (**b**) of antibiotic resistance genes (ARGs) among different treatments, heatmap of the dominant ARGs based on common logarithmic transformed abundance (**c**), and bipartite network showed the shared and unique ARGs types (**d**) among treatments. The nodes and edges were colored according to ARGs types. NG-CK, NG-CBD1, and NG-CBD2 represent the earthworm gut samples in the un-manured soil with 0, 1.0, and 2.0 mg kg^−1^ CBD, respectively. MG-CK, MG-CBD1, and MG-CBD2 represent the earthworm gut samples in the manured soil with 0, 1.0, and 2.0 mg kg^−1^ CBD, respectively

The total abundance of ARGs in the MG samples was significantly (*p <* 0.05) higher than that in the NG samples. Furthermore, the enhancement effect of CBD on the ARGs abundance in the earthworm gut was unraveled (Fig. 3A). The total abundance of ARGs was 0.039 copies per 16S-rRNA gene in the NG-CBD2 and 0.142 copies per 16S-rRNA gene in the MG-CBD2, which increased to 1.77-fold and 1.93-fold compared to the corresponding controls. The heatmap based on the logarithmic transformed abundance indicated that ARGs conferred resistance to several antibiotics which were mainly classified into 13 types (Fig. 3c). The abundance of some ARGs (i.e., multidrug, glycopeptide, tetracycline, rifamycin, MLSB, others, aminoglycoside, phenicol and sulfonamide resistance genes) in the NG-CBD2 was 1.67~2.46-fold higher than that in the control. CBD even at a low concentration (1 mg kg^−1^) increased the abundance of specific ARGs, such as rifamycin resistance genes. In terms of antibiotic resistance mechanism, CBD mainly enhanced the genes of antibiotic efflux pump and target alteration that accounted for the majority (*>* 70%) (Figure S7). The numbers of ARGs subtypes detected in the earthworm gut ranged from 30 to 155 with a bipartite network showing the shared and unique ARGs in Fig. 3d, and the MG-CBD2 treatment harbored the maximum unique ARGs (e.g., *aadA2*) while the dominant shared genes between treatments were multidrug ARGs. The diversity of ARGs significantly increased in the CBD2 treatment, compared to the controls (Fig. 3b, one-way ANOVA, *p <* 0.05).

**Fig. 4:**
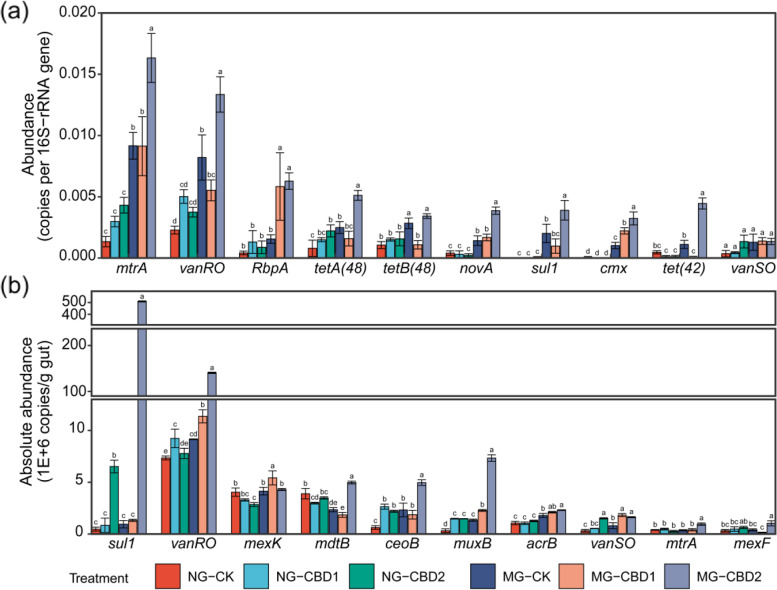
Abundance (**a**) and absolute abundance (**b**) of the dominant ARGs (top 10) in the earthworm gut among treatments. NG-CK, NG-CBD1, and NG-CBD2 represent the earthworm gut samples in the un-manured soil with 0, 1.0, and 2.0 mg kg^−1^ CBD, respectively. MG-CK, MG-CBD1, and MG-CBD2 represent the earthworm gut samples in the manured soil with 0, 1.0, and 2.0 mg kg^−1^ CBD, respectively

The enhancement effects of CBD on the abundance of the top 10 dominant ARGs are depicted in Fig. 4a. Remarkably, the abundance of *mtrA*, *vanRO*, *RbpA*, *tetA(48)*, *novA*, *sul1*, *cmx,* and *tet(42)* in the MG-CBD2 was 1.63~4.04-fold higher than that in the control (*p <* 0.05), respectively. A similar enhancement effect of CBD was also confirmed on the absolute abundance of the dominant ARGs using the qPCR method (Fig. 4b). The absolute abundance of *sul1*, *vanRO*, *mdtB*, *ceoB*, *muxB*, *vanSO*, *mtrA*, and *mexF* in the MG-CBD2 increased 2.00~457.19-fold than that in the control (*p <* 0.05), and notably, the *sul1* and *vanRO* soared to 5.1E+8 copies/g and 1.19E+8 copies/g in the MG-CBD2, respectively. The PCoA results also revealed that the gut resistome in the NG-CBD2 and MG-CBD2 were significantly different from the control along the *X*-axis (explaining 48.32% of total variance) (Figure S8a). Nevertheless, the abundance (Figure S9a) and diversity (Figure S9b) of ARGs were not significantly (*p <* 0.05) changed in the soil samples and the PCoA results showed no significant separation between the CBD treatments (Figure S8b).

### CBD broadened the range of bacterial hosts carring ARGs in the gut

**Fig. 5:**
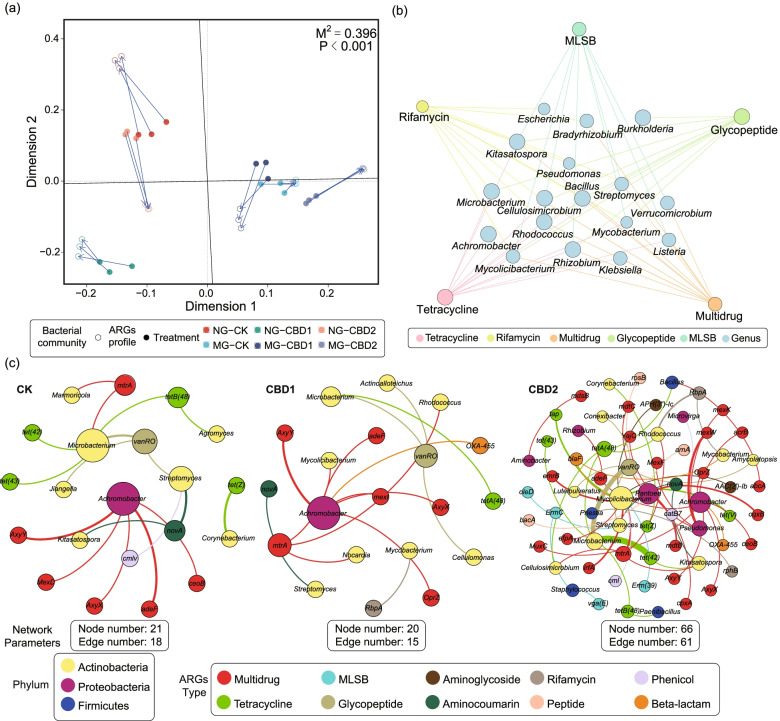
Procrustes analysis (**a**) of ARGs and bacterial communities, Spearman’s correlation-based co-occurrence network (**b**) of the dominant ARGs and genera (> 1%), and networks of ARGs hosts (**c**) based on the metagenomic assembly analysis in the earthworm gut among treatments. NG-CK, NG-CBD1, and NG-CBD2 represent the earthworm gut samples in the un-manured soil with 0, 1.0, and 2.0 mg kg^−1^ CBD, respectively. MG-CK, MG-CBD1, and MG-CBD2 represent the earthworm gut samples in the manured soil with 0, 1.0, and 2.0 mg kg^−1^ CBD, respectively

As shown in Fig. 5a, a significant correlation was observed between ARGs and bacterial communities in the earthworm gut using Procrustes analysis, exhibited by a goodness-of-fit test (*M*^2^ = 0.396, *p <* 0.001, 999 permutations). Several genera belonging to Actinobacteria (*Streptomyces*, *Microbacterium*, *Mycolicibacterium*, and *Kitasatospora*, etc.) were the most likely hosts of the dominant ARGs in the earthworm gut based on the Spearman’s correlation (Fig. 5b). And the multidrug resistance was the most shared ARG type involved 12 genera. Moreover, metagenomic assembly analysis was conducted to track the potential hosts of ARGs and result showed that CBD increased the diversity of bacterial hosts carrying ARGs in the earthworm gut (Fig. 5c). A total of 21 bacterial genera were assigned as the potential hosts of ARGs in the CBD2 treatment, among them, *Microbacterium* (*n* = 17) was the most frequent host of ARGs, including *tet(42)*, *vanRO*, *tetB(48)*, *tet(43)*, and *mtrA*, followed by *Mycolicibacterium* (*n* = 14), *Pantoea* (*n* = 11), *Achromobacter* (*n* = 8), and *Pseudomonas* (*n* = 6), almost of which belonged to phyla Actinobacteria and Proteobacteria. In addition, multidrug, tetracycline, and MLSB resistance genes (i.e., *mtrA, tet(42)*, and *ErmC*) were carried by multiple bacterial genera in the CBD2 treatment. However, only 8 bacterial genera were considered to carry ARGs in the control, and the constructed network was less complex than that in the CBD threatments. In addition, the potential hosts of ARGs in soil also mainly belonged to the phyla Proteobacteria and Actinobacteria, and some shared genera *Microbacterium* and *Pseudomonas* harboring ARGs were found between the earthworm gut and surrounding soil (Figure S10).

### CBD enhanced associations between ARGs and MGEs in the earthworm gut

**Fig. 6:**
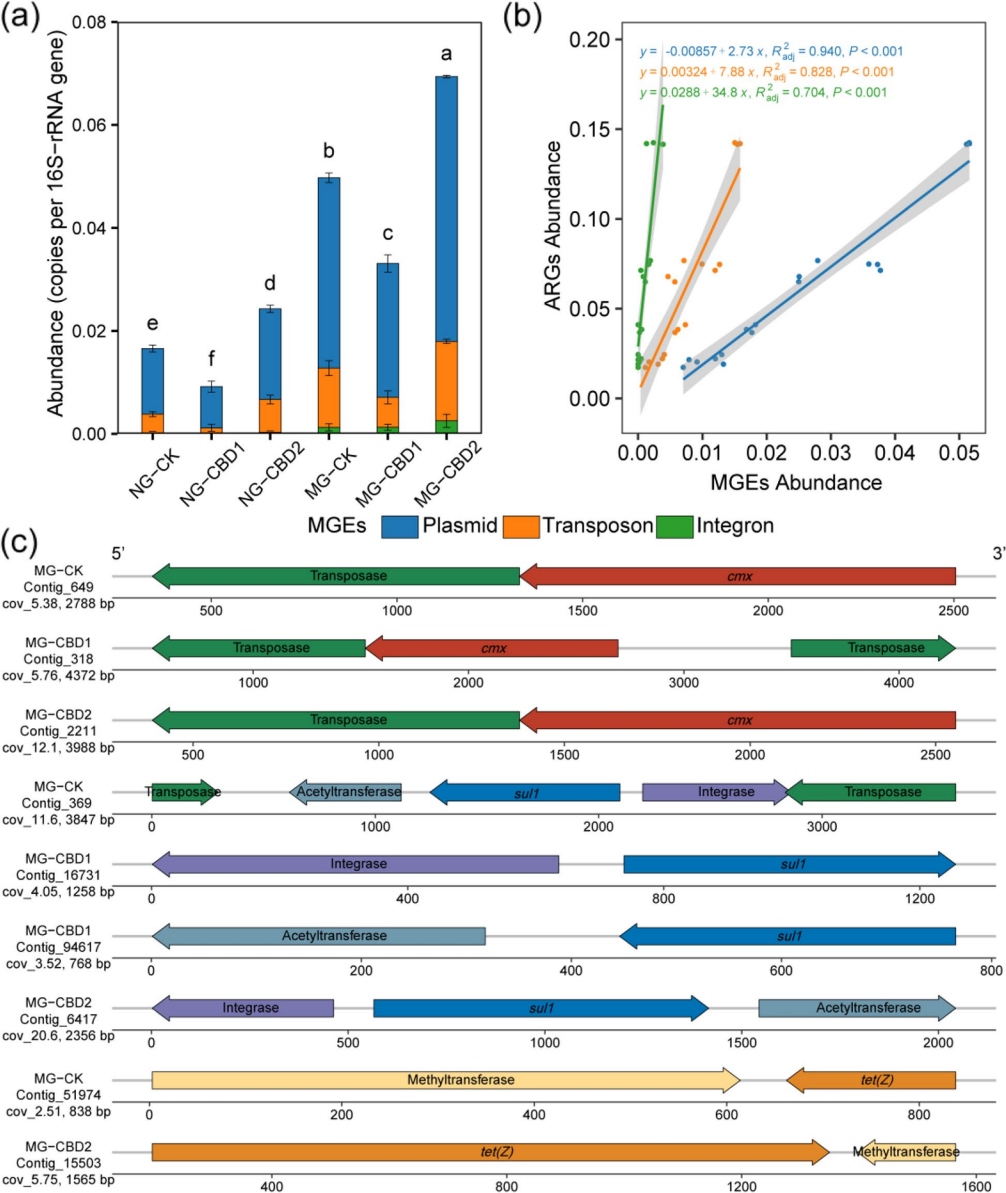
The abundance of MGEs (**a**), linear-regression analysis between the abundance of ARGs and MGEs (**b**), and the co-occurrence arrangements of ARGs and MGEs on the contigs (**c**) in the earthworm gut among treatments. NG-CK, NG-CBD1, and NG-CBD2 represent the earthworm gut samples in the un-manured soil with 0, 1.0, and 2.0 mg kg^−1^ CBD, respectively. MG-CK, MG-CBD1, and MG-CBD2 represent the earthworm gut samples in the manured soil with 0, 1.0, and 2.0 mg kg^−1^ CBD, respectively

As shown in Fig. 6a, both CBD exposure (2 mg kg^−1^) and manure addition could significantly (*p <* 0.05) increase the total abundance of MGEs in the earthworm gut. The abundance of plasmid, transposon, and integron was 1.38-, 1.55-, 1.82-fold higher than that in the control. Furthermore, Pearson’s correlation and linear regression analyses showed that the total abundance of ARGs was significantly positively correlated with the abundance of plasmid and transposon (*r* > 0.9, *p <* 0.001, Fig. 6b). In addition, more types of ARGs (e.g., MLSB, phenicol, and sulfonamide resistance genes) were positively correlated with MGEs (*p <* 0.01) in the CBD2 treatment than those in the other treatments (Figure S11). However, this positive correlation between ARGs and MGEs did not give a direct proof for the potential role of MGEs in ARGs dissemination. Herein, the co-occurrence arrangements of MGEs and ARGs in the contigs were also analyzed to reveal the potential of horizontal transfer of ARGs in the earthworm gut. As shown in Table S6, 11 pairs of co-occurrence patterns of ARGs and MGEs were found in the MG samples such as ARG-transposase and ARG-integrase, and the number and diversity of co-occurrence patterns in the MG-CBD2 were higher than those in the other treatments. Some representative co-occurrence patterns of ARGs and MGEs in the MG samples are presented in Fig. 6c. The *cmx*-transposase co-located on the same contig was shared in all treatments, and the co-occurrence patterns of *sul1-*integrase and *tet(Z)-*methyltransferase were also found, indicating a direct correlation between the abundance of ARGs and MGEs. In addition, several ARGs occurred on the contigs that belonged to the segments of various plasmids. As shown in Figure S12, the average percentage of plasmid-origin contigs carrying ARGs in the CBD2 treatment was 0.0040~0.0162%, which was 2.16~3.51-fold higher than that in the controls.

## Discussion

Earthworm is regarded as an important bioindicator for risk assessment due to its high bioaccumulation and sensitivity to pollutants [9, 33]. In this study, the BAF of CBD in the ME-CBD treatment was higher than that in the NE-CBD treatment, which may be due to that the manure addtion in the soil could alter the bioavailability of CBD to earthworm. The bioaccumulation potential of CBD by earthworm in soil may relate to soil physicochemical properties and earthworm species. Liu et al. (2012) reported that the bioavailability of CBD to earthworm in soil was influenced by soil physicochemical properties, such as organic matter and pH [7]**.** Some studies also found that different earthworm species exhibited various bioaccumulation abilities to CBD [34, 35].

In this study, applications of manure led to an anthropogenic introduction of resistome to not only agricultural soils but also the fauna gut, which was consistent with previous studies [36]. Twenty-eight days of CBD exposure at concentrations of 0~2.0 mg kg^−1^ in the soil was not obviously toxic to the earthworm, which was in keeping with the LC_50_ of CBD [34]. However, exposure to CBD significantly (*p <* 0.05) shaped resistome in the earthworm gut, and the enrichment of several dominant ARGs subtypes (e.g., *mtrA, vanRO, RbpA*, *sul1*, *tetA(48)*, and *cmx*) was observed. These observations implied CBD may serve as an important co-selective agent to aggravate antibiotic resistance in the gut. Several similar studies have also reported some polltants (e.g., antibiotics and heavy metals) could increase the abundance of ARGs in the fauna gut [12, 37]. For example, nonantibiotic carbamazepine accelerated antibiotic resistance in the collembolan gut, especially for the beta-lactams and multidrug resistance genes [13]. The enhanced ARGs were mainly related to the resistance mechanism of antibiotic efflux under CBD exposure. The multidrug efflux pump (e.g., RND superfamily) can actively extrude various toxic compounds, not only conventional antibiotics, but also non-antibiotic substrates such as heavy metals, pesticides, and dyes [38], so that the multidrug resistance genes (e.g., *mtrA*, *ceoB*, and *muxB*) were enhanced with the increasing concentrations of CBD. Besides, CBD residues also led to the increase of ARGs with other resistance mechanisms (e.g., *vanRO*, *sul1*, and *cmx*).

Meanwhile, based on the host-tracking results by metagenomic assembly analysis, resistome was harbored mainly by the bacterial hosts of Actinobacteria. Several studies have reported that CBD residues could alter the bacterial community structure [8], and our results showed that even a low concentration of CBD (1 mg kg^−1^) could significantly (*p <* 0.05) disturb the microbiota of the gut and soil. The changes in the gut microbiome were partially related to those in the soil. Several studies have reported that the gut microbiota of soil invertebrate including earthworm derived from soil microbial communities [39, 40]**.** In addition, some genera in the phylum Actinobacteria were enriched in the earthworm gut, which was similar to the effect of azoxystrobin on the *Enchytraeus crypticus* gut microbiome [41].

The increased relative abundance of Actinobacteria under CBD exposure might contribute to the increased ARGs in the earthworm gut. For example, the increased relative abundance of genus *Microbacterium* under CBD exposure may lead to an enhancement of some ARGs harbored in it (e.g., *vanRO*). A previous study reported that CBD could elevate the relative abundance of bacterial genera involved in 13 ARGs and increase the soil bacterial community resistance to chlortetracycline [8]. Some bacteria in the phylum Actinobacteria are known as the major antibiotic-producing microbes carrying multiple ARGs, and evidence showed that some ARGs harbored in the phylum Actinobacteria could be transferred to pathogens [42]. Simultaneously, Actinobacteria also play an important role in the metabolization of organic matter [43], the enrichment may contribute to keeping the earthworms healthy. The polymyxin B was reported that it could enrich Actinobacteria in the earthworm gut which helped earthworms adapt to the stress [44].

Furthermore, it is diffusely acknowledged that horizontal gene transfer via MGEs is a vital mechanism for ARGs dissemination [45, 46]. This study gives a novel insight into the potential facilitating effects of CBD on the ARGs dissemination mediated by MGEs in the earthworm gut using both metagenomic assembly method and correlation analysis. Similarly, heavy metals have been confirmed to promote MGEs-mediated ARGs transfer at environmental concentration [47]. Based on the assembled contigs, the co-occurrence patterns of *cmx*-transposase, *sul1*-integrase, and *tet(Z)*-methyltransferase were found prevalent in the MG samples, and CBD increased their number and diversity which may lead to the enhancement of these ARGs. The chloramphenicol exporter gene *cmx* was recently found that it transferred from Actinobacteria to clinical isolates of *Pseudomonas*, *Klebsiella*, and *Enterobacter* by the “carry-back” model [42], and *sul1* was frequently found to be linked with integrative conjugative elements such as integrons [46].

## Conclusions

The results obtained in this study showed that CBD could significantly shape the microbiome in the earthworm gut and enrich specific bacteria mainly belonging to the phylum Actinobacteria. Moreover, served as a co-selective agent, CBD could also elevate the abundance and diversity of ARGs in the earthworm gut. The enhancement effect of CBD on the resistome in the earthworm gut may be attributed to diverse potential bacterial hosts carring ARGs and facilitation on the ARGs dissemination mediated by MGEs. This study provides a novel insight into the neglected ecotoxicological risk of the widely used agrochemicals on the gut microbiome and resistome of the earthworm dwelling in soil.

## Supplementary Information

**Additional file 1: Table S1.** Physiochemical properties of the collected soils and chicken manure. **Table S2.** Information of metagenomic datasets in all samples. **Table S3.** Primers set of ARGs for qPCR. **Table S4.** Dissipation characteristics of CBD in the different treatments. **Table S5.** Survival number and rate of earthworms in the different treatments. **Table S6.** Co-occurrence patterns of ARGs and MGEs on the same contigs in the earthworm gut from the different treatments. **Figure S1.** Dissipation characteristics of CBD in the different treatments. **Figure S2.** Changes of earthworm biomass (fresh weight) among treatments. **Figure S3.** Shannon indices in the earthworm gut (a) and soil (b) in the different treatments. **Figure S4.** Heatmap of the dominant genera (Top 50) based on the common logarithm of relative abundance in the earthworm gut (a) and soil (b) from the different treatments. **Figure S5.** Variation in the relative abundance of the dominant genera (> 0.1%) mainly belonging to Proteobacteria and Actinobacteria in the earthworm gut. **Figure S6.** PCoA plot of bacterial communities in the earthworm gut (a) and soil (b) among treatments. **Figure S7.** Antibiotic resistance mechanism of ARGs in the earthworm gut from the different treatments. **Figure S8.** PCoA plot of ARGs profiles in the earthworm gut (a) and soil (b) among treatments. **Figure S9.** Comparison of total abundance (a) and diversity (b) of antibiotic resistance genes (ARGs) in the soil among treatments. **Figure S10.** Network of ARGs hosts based on the metagenomic assembly analysis in the soil. **Figure S11.** Pearson’s correlations between ARGs and MGEs in the earthworm gut among treatments. **Figure S12.** Percentage of plasmid-origin contigs carrying ARGs in the earthworm gut from the different treatments.

## Data Availability

Metagenomic sequencing data set used in this study was uploaded and the information is shown in Table S2. The laboratory experiment sequencing data has been deposited in the National Center for Biotechnology Information Sequence Read Archive (SRA) database (accession number: PRJNA773059).

## Abbreviations

ANOVA: Analysis of variance
ARGs: Antibiotic resistance genes
CBD: Carbendazim
MGEs: Mobile genetic elements
PCoA: Principal coordinates analysis

## References

[1] Rayne N, Aula L (2020). Livestock manure and the impacts on soil health: a review. Soil Syst.

[2] Zhu YG, Johnson TA, Su JQ, Qiao M, Guo GX, Stedtfeld RD, Hashsham SA, Tiedje JM (2013). Diverse and abundant antibiotic resistance genes in Chinese swine farms. Proc Natl Acad Sci U S A.

[3] Fang H, Wang H, Cai L, Yu Y (2015). Prevalence of antibiotic resistance genes and bacterial pathogens in long-term manured greenhouse soils as revealed by metagenomic survey. Environ Sci Technol.

[4] Wang Z, Di S, Qi P, Xu H, Zhao H, Wang X (2021). Dissipation, accumulation and risk assessment of fungicides after repeated spraying on greenhouse strawberry. Sci Total Environ.

[5] Singh S, Singh N, Kumar V, Datta S, Wani AB, Singh D, Singh K, Singh J (2016). Toxicity, monitoring and biodegradation of the fungicide carbendazim. Environ Chem Lett.

[6] Yan H, Wang D, Dong B, Tang F, Wang B, Fang H, Yu Y (2011). Dissipation of carbendazim and chloramphenicol alone and in combination and their effects on soil fungal:bacterial ratios and soil enzyme activities. Chemosphere.

[7] Liu K, Pan X, Han Y, Tang F, Yu Y (2012). Estimating the toxicity of the weak base carbendazim to the earthworm (Eisenia fetida) using in situ pore water concentrations in different soils. Sci Total Environ.

[8] Fang H, Han L, Cui Y, Xue Y, Cai L, Yu Y (2016). Changes in soil microbial community structure and function associated with degradation and resistance of carbendazim and chlortetracycline during repeated treatments. Sci Total Environ.

[9] Shi Z, Tang Z, Wang C (2017). A brief review and evaluation of earthworm biomarkers in soil pollution assessment. Environ Sci Pollut Res Int.

[10] Sun M, Chao H, Zheng X, Deng S, Ye M, Hu F (2020). Ecological role of earthworm intestinal bacteria in terrestrial environments: A review. Sci Total Environ.

[11] Yasmin S, D'Souza D (2007). Effect of pesticides on the reproductive output of Eisenia fetida. Bull Environ Contam Toxicol.

[12] Zhu D, An XL, Chen QL, Yang XR, Christie P, Ke X, Wu LH, Zhu YG (2018). Antibiotics disturb the microbiome and increase the incidence of resistance genes in the gut of a common soil collembolan. Environ Sci Technol.

[13] Wang YF, Qiao M, Zhu D, Zhu YG (2020). Antibiotic resistance in the collembolan gut microbiome accelerated by the nonantibiotic drug carbamazepine. Environ Sci Technol.

[14] Wang HT, Chi QQ, Zhu D, Li G, Ding J, An XL, Zheng F, Zhu YG, Xue XM (2019). Arsenic and sulfamethoxazole increase the incidence of antibiotic resistance genes in the gut of earthworm. Environ Sci Technol.

[15] Fang H, Han L, Zhang H, Long Z, Cai L, Yu Y (2018). Dissemination of antibiotic resistance genes and human pathogenic bacteria from a pig feedlot to the surrounding stream and agricultural soils. J Hazard Mater.

[16] Zhang H, Chen S, Zhang Q, Long Z, Yu Y, Fang H (2020). Fungicides enhanced the abundance of antibiotic resistance genes in greenhouse soil. Environ Pollut.

[17] Chen S, Zhou Y, Chen Y, Gu J (2018). fastp: an ultra-fast all-in-one FASTQ preprocessor. Bioinformatics.

[18] Langmead B, Salzberg SL (2012). Fast gapped-read alignment with Bowtie 2. Nat Methods.

[19] Lu J, Breitwieser FP, Thielen P, Salzberg SL (2017). Bracken: estimating species abundance in metagenomics data. PeerJ Comput Sci.

[20] Wood DE, Lu J, Langmead B (2019). Improved metagenomic analysis with Kraken 2. Genome Biol.

[21] Alcock BP, Raphenya AR, Lau TTY, Tsang KK, Bouchard M, Edalatmand A, Huynh W, Nguyen AV, Cheng AA, Liu S (2020). CARD 2020: antibiotic resistome surveillance with the comprehensive antibiotic resistance database. Nucleic Acids Res.

[22] Parnanen K, Karkman A, Hultman J, Lyra C, Bengtsson-Palme J, Larsson DGJ, Rautava S, Isolauri E, Salminen S, Kumar H (2018). Maternal gut and breast milk microbiota affect infant gut antibiotic resistome and mobile genetic elements. Nat Commun.

[23] Li B, Yang Y, Ma L, Ju F, Guo F, Tiedje JM, Zhang T (2015). Metagenomic and network analysis reveal wide distribution and co-occurrence of environmental antibiotic resistance genes. ISME J.

[24] Zhang H, Zhang Q, Chen S, Zhang Z, Song J, Long Z, Yu Y, Fang H (2020). Enterobacteriaceae predominate in the endophytic microbiome and contribute to the resistome of strawberry. Sci Total Environ.

[25] Nurk S, Meleshko D, Korobeynikov A, Pevzner PA (2017). metaSPAdes: a new versatile metagenomic assembler. Genome Res.

[26] Hyatt D, Chen GL, Locascio PF, Land ML, Larimer FW, Hauser LJ (2010). Prodigal: prokaryotic gene recognition and translation initiation site identification. BMC Bioinformatics.

[27] Forsberg KJ, Patel S, Gibson MK, Lauber CL, Knight R, Fierer N, Dantas G (2014). Bacterial phylogeny structures soil resistomes across habitats. Nature.

[28] Buchfink B, Reuter K, Drost HG (2021). Sensitive protein alignments at tree-of-life scale using DIAMOND. Nat Methods.

[29] Zhao R, Yu K, Zhang J, Zhang G, Huang J, Ma L, Deng C, Li X, Li B (2020). Deciphering the mobility and bacterial hosts of antibiotic resistance genes under antibiotic selection pressure by metagenomic assembly and binning approaches. Water Res.

[30] Menzel P, Ng KL, Krogh A (2016). Fast and sensitive taxonomic classification for metagenomics with Kaiju. Nat Commun.

[31] Pellow D, Mizrahi I, Shamir R (2020). PlasClass improves plasmid sequence classification. Plos Comput Biol.

[32] Bastian M, Heymann S, Jacomy M (2009). Gephi: an open source software for exploring and manipulating networks. Third Int ICWSM Conf.

[33] Vischetti C, Casucci C, De Bernardi A, Monaci E, Tiano L, Marcheggiani F (1892). Sub-lethal effects of pesticides on the DNA of soil organisms as early ecotoxicological biomarkers. Front Microbiol.

[34] Daam MA, Garcia MV, Scheffczyk A, Rombke J (2020). Acute and chronic toxicity of the fungicide carbendazim to the earthworm Eisenia fetida under tropical versus temperate laboratory conditions. Chemosphere.

[35] Burrows LA, Edwards CA (2004). The use of integrated soil microcosms to assess the impact of carbendazim on soil ecosystems. Ecotoxicology.

[36] Ding J, Zhu D, Hong B, Wang HT, Li G, Ma YB, Tang YT, Chen QL (2019). Long-term application of organic fertilization causes the accumulation of antibiotic resistome in earthworm gut microbiota. Environ Int.

[37] Ding J, An XL, Lassen SB, Wang HT, Zhu D, Ke X (2019). Heavy metal-induced co-selection of antibiotic resistance genes in the gut microbiota of collembolans. Sci Total Environ.

[38] Blanco P, Hernando-Amado S, Reales-Calderon JA, Corona F, Lira F, Alcalde-Rico M, Bernardini A, Sanchez MB, Martinez JL (2016). Bacterial multidrug efflux pumps: much more than antibiotic resistance determinants. Microorganisms.

[39] Berg M, Stenuit B, Ho J, Wang A, Parke C, Knight M, Alvarez-Cohen L, Shapira M (2016). Assembly of the Caenorhabditis elegans gut microbiota from diverse soil microbial environments. ISME J.

[40] Drake HL, Horn MA (2007). As the worm turns: the earthworm gut as a transient habitat for soil microbial biomes. Annu Rev Microbiol.

[41] Zhang Q, Zhu D, Ding J, Zheng F, Zhou S, Lu T, Zhu YG, Qian H (2019). The fungicide azoxystrobin perturbs the gut microbiota community and enriches antibiotic resistance genes in Enchytraeus crypticus. Environ Int.

[42] Jiang X, Ellabaan MMH, Charusanti P, Munck C, Blin K, Tong Y, Weber T, Sommer MOA, Lee SY (2017). Dissemination of antibiotic resistance genes from antibiotic producers to pathogens. Nat Commun.

[43] Long Z, Wang X, Wang Y, Dai H, Li C, Xue Y (2021). Characterization of a novel carbendazim-degrading strain Rhodococcus sp. CX-1 revealed by genome and transcriptome analyses. Sci Total Environ.

[44] Li L, Zhu D, Yi X, Su J, Duan G, Tang X, Zhu Y (2021). Combined pollution of arsenic and Polymyxin B enhanced arsenic toxicity and enriched ARG abundance in soil and earthworm gut microbiotas. J Environ Sci (China).

[45] Fang H, Lian J, Wang H, Cai L, Yu Y (2015). Exploring bacterial community structure and function associated with atrazine biodegradation in repeatedly treated soils. J Hazard Mater.

[46] Gillings MR, Gaze WH, Pruden A, Smalla K, Tiedje JM, Zhu YG (2015). Using the class 1 integron-integrase gene as a proxy for anthropogenic pollution. ISME J.

[47] Lu J, Wang Y, Jin M, Yuan Z, Bond P, Guo J (2020). Both silver ions and silver nanoparticles facilitate the horizontal transfer of plasmid-mediated antibiotic resistance genes. Water Res.

