# Supplementary material for: Carbendazim shapes microbiome and enhances resistome in the earthworm gut
Source: Microbiome. 2022 Apr 18;10:63. doi: 10.1186/s40168-022-01261-8 (PMC9014604; doi:10.1186/s40168-022-01261-8)
Supplement: Supplementary file 2 — Additional file 1: Table S1. Physiochemical properties of the collected soils and chicken manure. Table S2. Information of metagenomic datasets in all samples. Table S3. Primers set of ARGs for qPCR. Table S4. Dissipation characteristics of CBD in the different treatments. Table S5. Survival number and rate of earthworms in the different treatments. Table S6. Co-occurrence patterns of ARGs and MGEs on the same contigs in the earthworm gut from the different treatments. Figure S1. Dissipation characteristics of CBD in the different treatments. Figure S2. Changes of earthworm biomass (fresh weight) among treatments. Figure S3. Shannon indices in the earthworm gut (a) and soil (b) in the different treatments. Figure S4. Heatmap of the dominant genera (Top 50) based on the common logarithm of relative abundance in the earthworm gut (a) and soil (b) from the different treatments. Figure S5. Variation in the relative abundance of the dominant genera (> 0.1%) mainly belonging to Proteobacteria and Actinobacteria in the earthworm gut. Figure S6. PCoA plot of bacterial communities in the earthworm gut (a) and soil (b) among treatments. Figure S7. Antibiotic resistance mechanism of ARGs in the earthworm gut from the different treatments. Figure S8. PCoA plot of ARGs profiles in the earthworm gut (a) and soil (b) among treatments. Figure S9. Comparison of total abundance (a) and diversity (b) of antibiotic resistance genes (ARGs) in the soil among treatments. Figure S10. Network of ARGs hosts based on the metagenomic assembly analysis in the soil. Figure S11. Pearson’s correlations between ARGs and MGEs in the earthworm gut among treatments. Figure S12. Percentage of plasmid-origin contigs carrying ARGs in the earthworm gut from the different treatments. [file 40168_2022_1261_MOESM2_ESM.docx]

Supplementary Information

**Carbendazim shapes microbiome and enhances resistome in the earthworm gut**

Jiajin Song^a^, Tongxin Li^a^, Zhiruo Zheng^a^, Wenjie Fu^d^, Zhengnan Long^a^, Nan Shi^b^, Yuling Han^c^, Luqing Zhang^a^, Yunlong Yu^a^, Hua Fang*^, a^

^a^*Institute of Pesticide and Environmental Toxicology,* *College of Agriculture and Biotechnology, Zhejiang University, Hangzhou 310058, China*

^b^*Department of Developmental and Cell Biology, University of California, Irvine CA 92697, United States*

^c^*Institue of Environmental Health and Pollution Control, School of Environmental Science and Engineering, Guangdong University of Technology, Guangzhou 510006, China*

^d^*Institute of Insect Sciences, College of Agriculture and Biotechnology, Zhejiang University, Hangzhou 310058, China*

*Correspondence author, Tel/Fax: +86-571-88982810, E-mail: agri@zju.edu.cn

**Contents**

**Tables**

**Table S1.** Physiochemical properties of the collected soils and chicken manure.

**Table S2.** Information of metagenomic datasets in all samples.

**Table S3.** The primers set of ARGs for qPCR.

**Table S4.** Dissipation characteristics of CBD in the different treatments.

**Table S5.** Survival number and rate of earthworms in the different treatments.

**Table S6.** The co-occurrence patterns of ARGs and MGEs on the same contigs in the earthworm gut from the different treatments.

**Figures**

**Figure S1.** Dissipation characteristics of CBD in the different treatments.

**Figure S2.** Changes of earthworm biomass (fresh weight) among treatments.

**Figure S3.** Shannon indices in the earthworm gut (a) and soil (b) in the different treatments.

**Figure S4.** Heatmap of the dominant genera (Top 50) based on the common logarithm of relative abundance in the earthworm gut (a) and soil (b) from the different treatments.

**Figure S5.** Variation in the relative abundance of the dominant genera (>0.1%) mainly belonging to Proteobacteria and Actinobacteria in the earthworm gut.

**Figure S6.** PCoA plot of bacterial communities in the earthworm gut (a) and soil (b) among treatments.

**Figure S7.** Antibiotic resistance mechanism of ARGs in the earthworm gut from the different treatments.

**Figure S8.** PCoA plot of ARGs profiles in the earthworm gut (a) and soil (b) among treatments.

**Figure S9.** Comparison of total abundance (a) and diversity (b) of antibiotic resistance genes (ARGs) in the soil among treatments.

**Figure S10.** Network of ARGs hosts based on the metagenomic assembly analysis in the soil.

**Figure S11.** Pearson's correlations between ARGs and MGEs in the earthworm gut among treatments.

**Figure S12.** Percentage of plasmid-origin contigs carrying ARGs in the earthworm gut from the different treatments.

**Total:**

**Number of tables: 6**

**Number of figures: 12**

**Number of pages: 22**

**Table S1.** Physiochemical properties of the collected soil and chicken manure.

| Type | Textures | pH | OM (%) | Total N (%) | CEC (cmol·kg^-1)^ | Sand (%) | Silt (%) | Clay (%) |
| --- | --- | --- | --- | --- | --- | --- | --- | --- |
| Soil | Silty clay loam | 6.8 | 3.1 | 0.14 | 10.6 | 21.5 | 71.1 | 7.4 |
| Chicken manure | / | 7.2 | 66 | 2.5 | / | / | / | / |

*OM, organic matter; Total N, total nitrogen; CEC, cation exchange capacity.

**Table S2.** Information of metagenomic datasets in all samples.

| **Samples** | **Samples_ID** | **Raw**  **pair-end reads number** | **Clean reads number**  **after 'fastp' treatment** | **Not aligned to earthworm reads number**  **after 'bowtie2' treatment** |
| --- | --- | --- | --- | --- |
| NG-CK | NG-CK-1 | 16,422,630 | 16,169,412 | 8,923,336 |
|  | NG-CK-2 | 15,690,888 | 15,449,254 | 8,527,504 |
|  | NG-CK-3 | 16,241,598 | 15,990,214 | 8,833,008 |
| MG-CK | MG-CK-1 | 19,541,458 | 19,214,738 | 12,086,676 |
|  | MG-CK-2 | 20,900,592 | 20,551,170 | 12,925,610 |
|  | MG-CK-3 | 18,718,402 | 18,406,234 | 11,576,544 |
| NG-CBD1 | NG-CBD1-1 | 20,864,134 | 20,538,340 | 11,524,066 |
|  | NG-CBD1-2 | 20,231,574 | 19,913,518 | 11,179,334 |
|  | NG-CBD1-3 | 21,214,586 | 20,881,296 | 11,720,984 |
| MG-CBD1 | MG-CBD1-1 | 20,379,248 | 19,968,620 | 12,556,664 |
|  | MG-CBD1-2 | 19,645,678 | 19,249,564 | 12,103,510 |
|  | MG-CBD1-3 | 20,130,330 | 19,725,504 | 12,402,002 |
| NG-CBD2 | NG-CBD2-1 | 18,739,076 | 18,390,494 | 11,648,968 |
|  | NG-CBD2-2 | 19,494,854 | 19,132,276 | 12,118,412 |
|  | NG-CBD2-3 | 18,942,704 | 18,588,868 | 11,772,968 |
| MG-CBD2 | MG-CBD2-1 | 16,299,522 | 15,924,978 | 12,425,728 |
|  | MG-CBD2-2 | 16,518,860 | 16,136,964 | 12,591,886 |
|  | MG-CBD2-3 | 17,036,502 | 16,642,948 | 12,984,658 |
| NS-CK | NS-CK-1 | 12,757,298 | 12,545,394 | / |
|  | NS-CK-2 | 13,033,856 | 12,816,254 | / |
|  | NS-CK-3 | 12,249,112 | 12,044,614 | / |
| MS-CK | MS-CK-1 | 12,520,874 | 12,204,516 | / |
|  | MS-CK-2 | 13,403,066 | 13,063,012 | / |
|  | MS-CK-3 | 12,827,930 | 12,502,728 | / |
| NS-CBD1 | NS-CBD1-1 | 12,293,386 | 12,038,508 | / |
|  | NS-CBD1-2 | 11,849,028 | 11,602,126 | / |
|  | NS-CBD1-3 | 12,401,188 | 12,142,598 | / |
| MS-CBD1 | MS-CBD1-1 | 13,138,534 | 12,907,602 | / |
|  | MS-CBD1-2 | 12,337,546 | 12,121,004 | / |
|  | MS-CBD1-3 | 13,123,564 | 12,892,884 | / |
| NS-CBD2 | NS-CBD2-1 | 12,853,960 | 12,647,372 | / |
|  | NS-CBD2-2 | 12,583,618 | 12,381,196 | / |
|  | NS-CBD2-3 | 11,919,862 | 11,727,894 | / |
| MS-CBD2 | MS-CBD2-1 | 13,011,970 | 12,779,014 | / |
|  | MS-CBD2-2 | 12,721,982 | 12,493,576 | / |
|  | MS-CBD2-3 | 13,351,604 | 13,112,756 | / |

*NG-CK, NG-CBD1, and NG-CBD2 represent the earthworm gut samples in the un-manured soil with 0, 1.0, and 2.0 mg·kg^−1^ CBD, respectively. MG-CK, MG-CBD1, and MG-CBD2 represent the earthworm gut samples in the manured soil with 0, 1.0, and 2.0 mg·kg^−1^ CBD, respectively. NS-CK, NS-CBD1, and NS-CBD2 represent the soil samples in the un-manured soil with 0, 1.0, and 2.0 mg·kg^−1^ CBD, respectively. MS-CK, MS-CBD1, and MS-CBD2 represent the soil samples in the manured soil with 0, 1.0, and 2.0 mg·kg^−1^ CBD, respectively.

**Table S3.** The primers set of ARGs for qPCR.

| **ARG name** | **Primer sequence** | | **Length (bp)** | **Annealing temperature (℃)** | **Standard curve** | **Correlation coefficient** | **Amplification efficiency**  **(%)** |
| --- | --- | --- | --- | --- | --- | --- | --- |
| *acrB* | F | TGGTGCTATCTATCGTCAGTTCTCT | 268 | 62 | y = -3.19x + 43.64 | 0.9963 | 105.82 |
|  | R | CATGCCGACCACGATGATCAG |  |  |  |  |  |
| *ceoB* | F | AAGTGAGCGGGCCGATCATC | 262 | 64 | y = -3.78x + 46.35 | 0.9923 | 83.89 |
|  | R | TTGTTGAAGCCGCGGAAGAA |  |  |  |  |  |
| *muxB* | F | GACTGGCAGGACAAGGGCTT | 312 | 61 | y = -3.34x + 43.38 | 0.9989 | 99.25 |
|  | R | GAACTGGGCGATATGGTTGATC |  |  |  |  |  |
| *mexF* | F | GATCCTCGCCGGCAATACCG | 270 | 65 | y = -3.55x + 39.95 | 0.9995 | 91.29 |
|  | R | CCTTCCTCCTGCTTGTCCTTGG |  |  |  |  |  |
| *mexK* | F | GGTGAAGATGGAGCAGGGCTA | 225 | 61 | y = -3.16x + 42.24 | 0.9909 | 107.23 |
|  | R | CCCAGGTAGGGAACGAAGACC |  |  |  |  |  |
| *vanRO* | F | GCCCCTCCGGTGACGAGAT | 167 | 65 | y = -3.42x + 41.71 | 0.9936 | 96.06 |
|  | R | ACGGAGCACGAGCTCCTGCA |  |  |  |  |  |
| *vanSO* | F | TTGCGGCGGTTTGGGTTTTC | 256 | 65 | y = -3.81x + 44.36 | 0.9905 | 83.01 |
|  | R | CGGTACGACAGCGAGCCACT |  |  |  |  |  |
| *mtrA* | F | CATCGACGTGTGCCGGGTGT | 255 | 64 | y = -3.72x + 45.42 | 0.9968 | 85.7 |
|  | R | CCGTTGCGAGTGACCTTGTG |  |  |  |  |  |
| *mdtB* | F | GGCATTCTGTACGAGAGCTTTATTCAC | 272 | 65 | y = -3.62x + 42.44 | 0.9964 | 88.9 |
|  | R | GTGGTCATCAGGATCGGACGAAA |  |  |  |  |  |
| *sul1* | F | GCGTGGGCTACCTGAACGAT | 226 | 66 | y = -3.46x + 39.71 | 0.9916 | 94.54 |
|  | R | ATGAGCCGGTCGGCAGCGAC |  |  |  |  |  |

**Table S4.** Dissipation characteristics of CBD in the different treatments.

| Treatment | Kinetic equation | Half-life (d) | R |
| --- | --- | --- | --- |
| NS-CBD1 | R = 0.84e^-0.0210t^ | 33.00 a | 0.9402 |
| MS-CBD1 | R = 0.90e^-0.0295t^ | 23.49 b | 0.9594 |
| NS-CBD2 | R = 1.76e^-0.0209t^ | 33.16 a | 0.9499 |
| MS-CBD2 | R = 1.87e^-0.0362t^ | 19.15 c | 0.9936 |

* NS-CBD1, NS-CBD2 represent the soil samples in the un-manured soil with 1.0 and 2.0 mg·kg^−1^ CBD, respectively. MS-CBD1 and MS-CBD2 represent the soil samples in the manured soil with 1.0 and 2.0 mg·kg^−1^ CBD, respectively.

**Table S5.** Survival number and rate of earthworms in the different treatments.

| Treatment | Initial earthworm number | Survival number | | | Survival rate（%） |
| --- | --- | --- | --- | --- | --- |
|  |  | 1 | 2 | 3 |  |
| NE-CK | 20 | 19 | 16 | 17 | 86.67 a |
| NE-CBD1 |  | 17 | 18 | 15 | 83.33 a |
| NE-CBD2 |  | 17 | 16 | 16 | 81.67 a |
| ME-CK |  | 18 | 18 | 17 | 88.33 a |
| ME-CBD1 |  | 17 | 19 | 14 | 83.33 a |
| ME-CBD2 |  | 16 | 17 | 15 | 80.00 a |

*Different lowercase letters indicate significant differences between different treatments according to one-way analysis of variance with least significant difference’s multiple range tests (p < 0.05). NE-CK, NE-CBD1, and NE-CBD2 represent the earthworm samples in the un-manured soil with 0, 1.0, and 2.0 mg·kg^−1^ CBD, respectively. ME-CK, ME-CBD1, and ME-CBD2 represent the earthworm samples in the manured soil with 0, 1.0, and 2.0 mg·kg^−1^ CBD, respectively.

**Table S6.** The co-occurrence patterns of ARGs and MGEs on the same contigs in the earthworm gut from the different treatments.

| **Sample** | **Contig_ID** | **Length (bp)** | **ARG** | **MGE** |
| --- | --- | --- | --- | --- |
| MG-CK | Contig_369 | 3847 | *sul1* | transposase, integrase, acetyltransferase |
| MG-CK | Contig_51974 | 838 | *tet(Z)* | methyltransferase |
| MG-CK | Contig_649 | 2788 | *cmx* | transposase |
| MG-CBD1 | Contig_16731 | 1258 | *sul1* | integrase |
| MG-CBD1 | Contig_318 | 4372 | *cmx* | transposase |
| MG-CBD1 | Contig_94617 | 768 | *sul1* | acetyltransferase |
| MG-CBD2 | Contig_1372 | 5003 | *ErmC* | recombinase |
| MG-CBD2 | Contig_15503 | 1565 | *tet(Z)* | methyltransferase |
| MG-CBD2 | Contig_2211 | 3988 | *cmx* | transposase |
| MG-CBD2 | Contig_5797 | 2479 | *fexA* | transposase |
| MG-CBD2 | Contig_6417 | 2356 | *sul1* | integrase, acetyltransferase |

*MG-CK, MG-CBD1, and MG-CBD2 represent the earthworm gut samples in the manured soil with 0, 1.0, and 2.0 mg·kg^−1^ CBD, respectively.


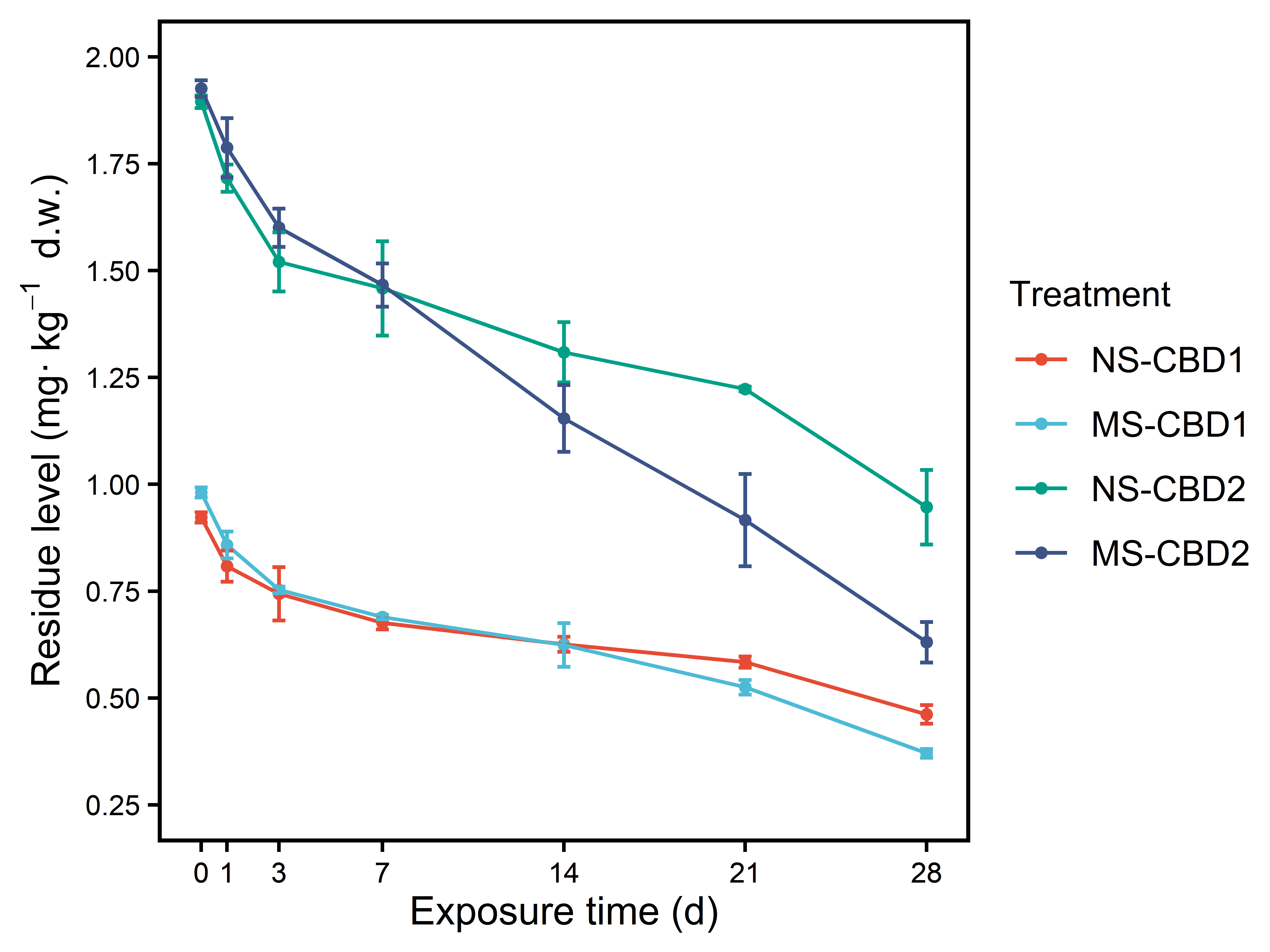


**Figure S1.** Dissipation characteristics of CBD in the different treatments. For different treatments, NS-CBD1, and NS-CBD2 represent the soil samples in the un-manured soil with 1.0 and 2.0 mg·kg^−1^ CBD, respectively. MS-CBD1, and MS-CBD2 represent the soil samples in the manured soil with 1.0 and 2.0 mg·kg^−1^ CBD treatment, respectively.


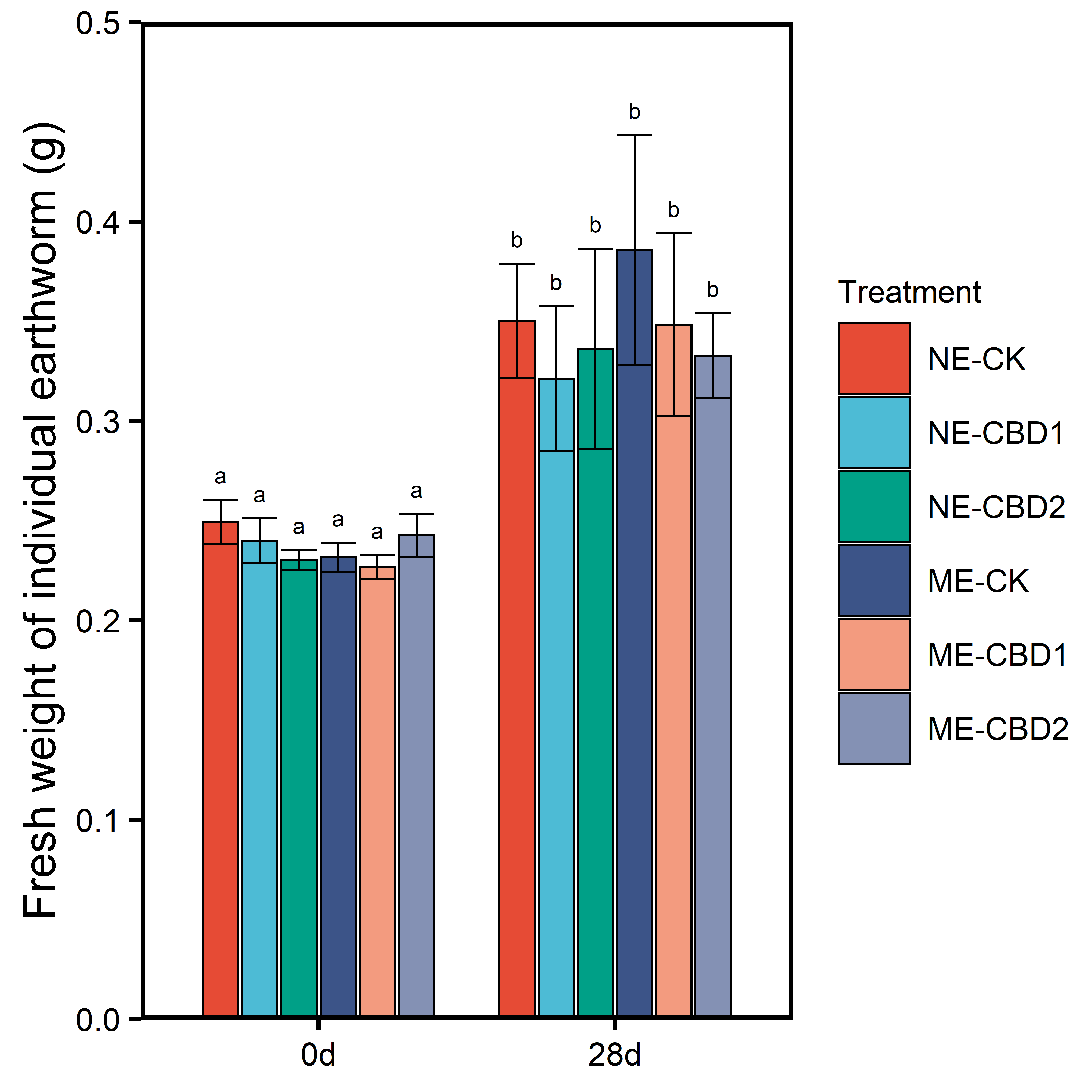


**Figure S2.** Changes of earthworm biomass (fresh weight) among treatments. Different lowercase letters indicate significant differences among treatments according to one-way analysis of variance with least significant difference’s multiple range tests (p < 0.05). NE-CK, NE-CBD1, and NE-CBD2 represent the earthworm samples in the un-manured soil with 0, 1.0, and 2.0 mg·kg^−1^ CBD, respectively. ME-CK, ME-CBD1, and ME-CBD2 represent the earthworm samples in the manured soil with 0, 1.0, and 2.0 mg·kg^−1^ CBD, respectively.


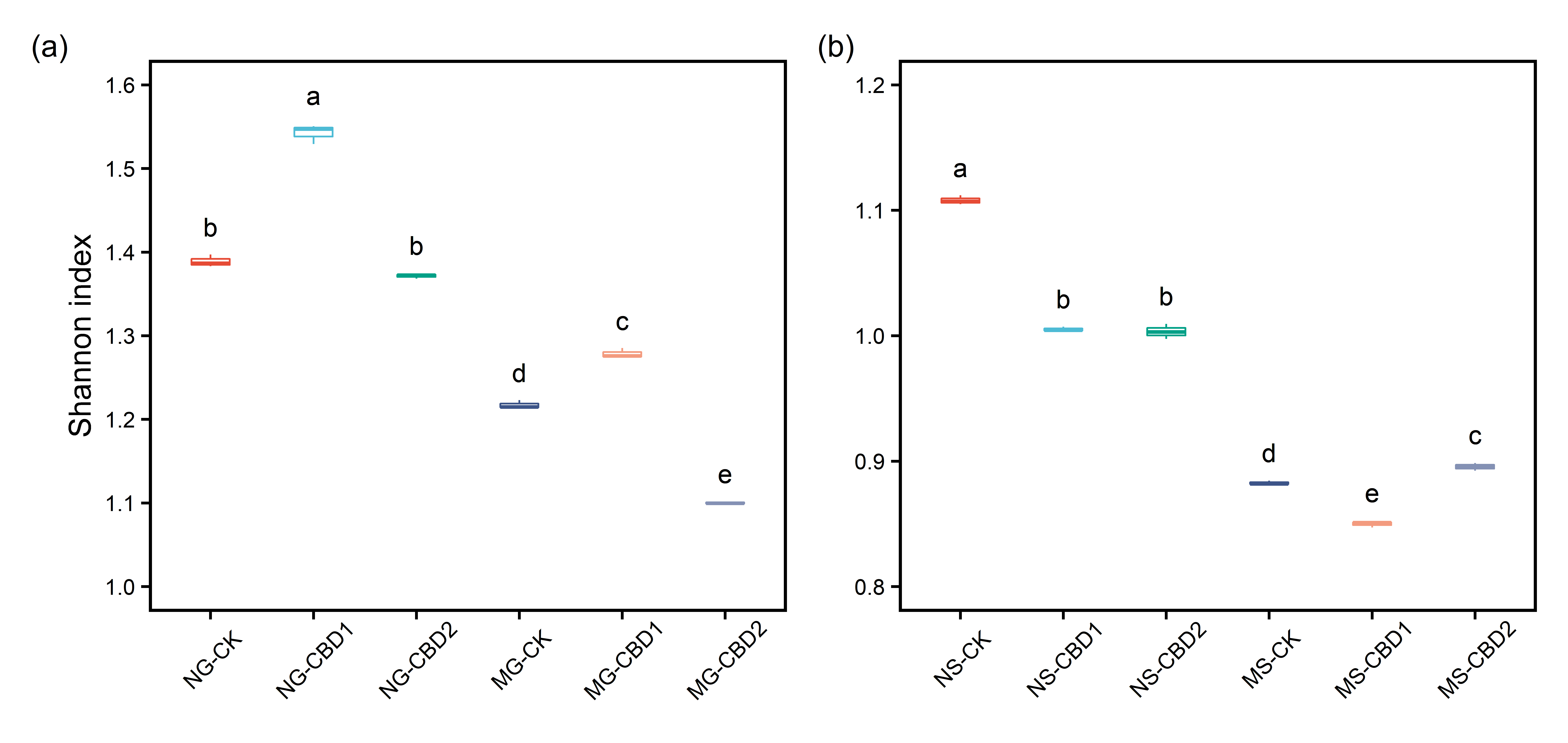


**Figure S3.** Shannon indices in the earthworm gut (a) and soil (b) in the different treatments. NG-CK, NG-CBD1, and NG-CBD2 represent the earthworm gut samples in the un-manured soil with 0, 1.0, and 2.0 mg·kg^−1^ CBD, respectively. MG-CK, MG-CBD1, and MG-CBD2 represent the earthworm gut samples in the manured soil with 0, 1.0, and 2.0 mg·kg^−1^ CBD, respectively. NS-CK, NS-CBD1, and NS-CBD2 represent the soil samples in the un-manured soil with 0, 1.0, and 2.0 mg·kg^−1^ CBD, respectively. MS-CK, MS-CBD1, and MS-CBD2 represent the soil samples in the manured soil with 0, 1.0, and 2.0 mg·kg^−1^ CBD, respectively.


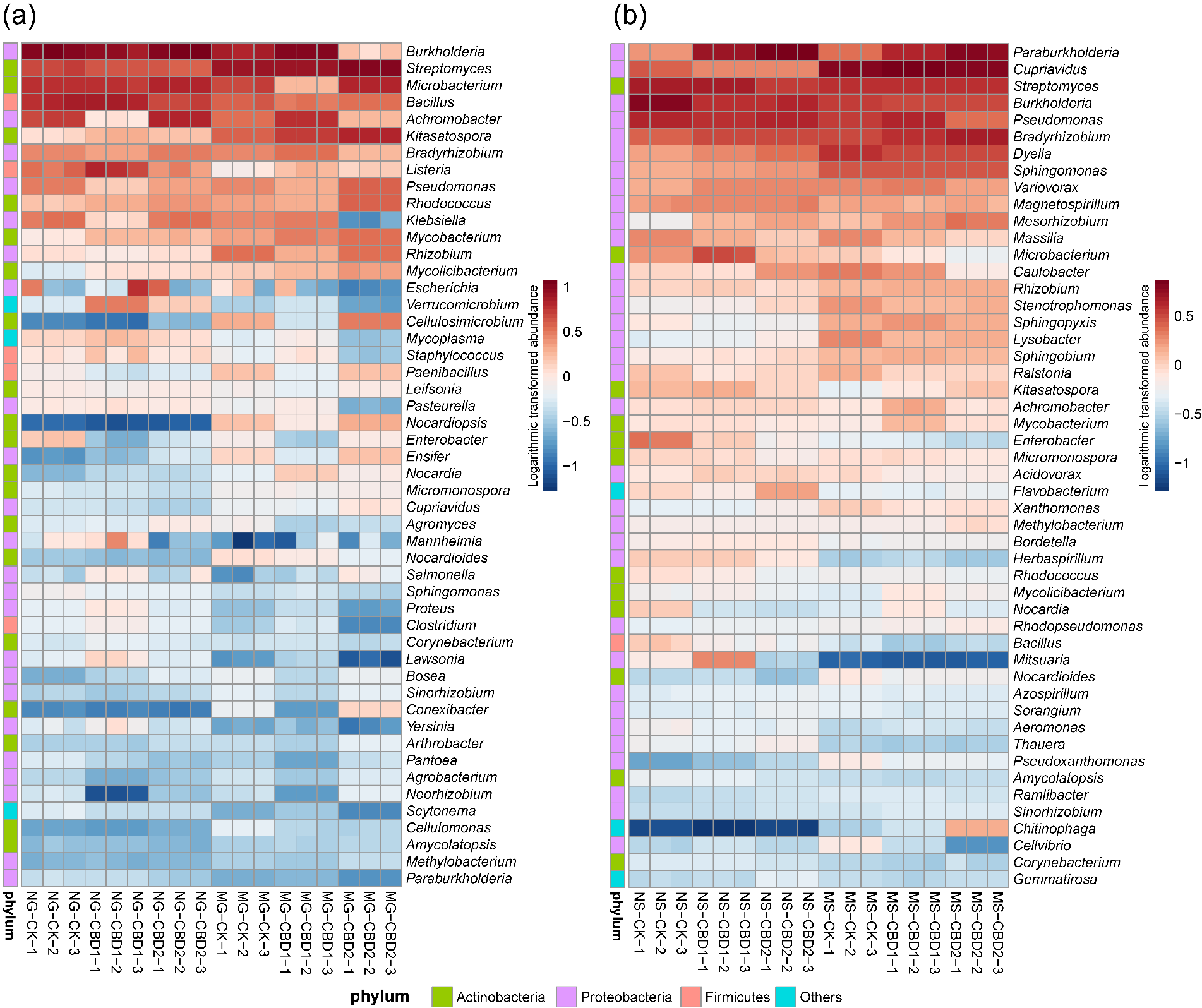


**Figure S4.** Heatmap of the dominant genera (Top 50) based on the common logarithm of relative abundance in the earthworm gut (a) and soil (b) from the different treatments. NG-CK, NG-CBD1, and NG-CBD2 represent the earthworm gut samples in the un-manured soil with 0, 1.0, and 2.0 mg·kg^−1^ CBD treatment, respectively. MG-CK, MG-CBD1, and MG-CBD2 represent the earthworm gut samples in the manured soil with 0, 1.0, and 2.0 mg·kg^−1^ CBD treatment, respectively. NS-CK, NS-CBD1, and NS-CBD2 represent the soil samples in the un-manured soil with 0, 1.0, and 2.0 mg·kg^−1^ CBD, respectively. MS-CK, MS-CBD1, and MS-CBD2 represent the soil samples in the manured soil with 0, 1.0, and 2.0 mg·kg^−1^ CBD, respectively.


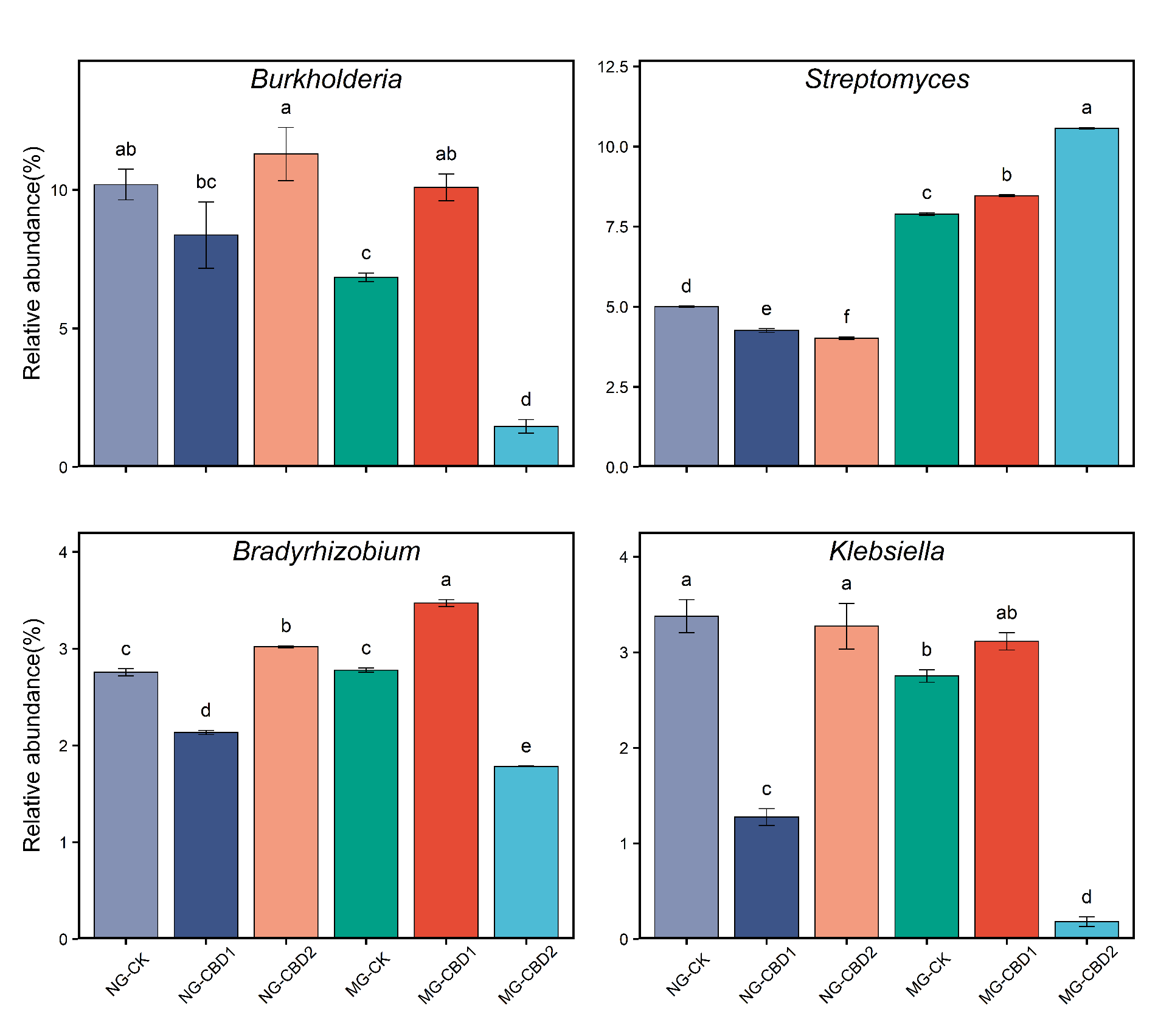


**Figure S5.** Variation in the relative abundance of the dominant genera (>0.1%) mainly belonging to Proteobacteria and Actinobacteria in the earthworm gut. NG-CK, NG-CBD1, and NG-CBD2 represent the earthworm gut samples in the un-manured soil with 0, 1.0, and 2.0 mg·kg^−1^ CBD treatment, respectively. MG-CK, MG-CBD1, and MG-CBD2 represent the earthworm gut samples in the manured soil with 0, 1.0, and 2.0 mg·kg^−1^ CBD treatment, respectively.


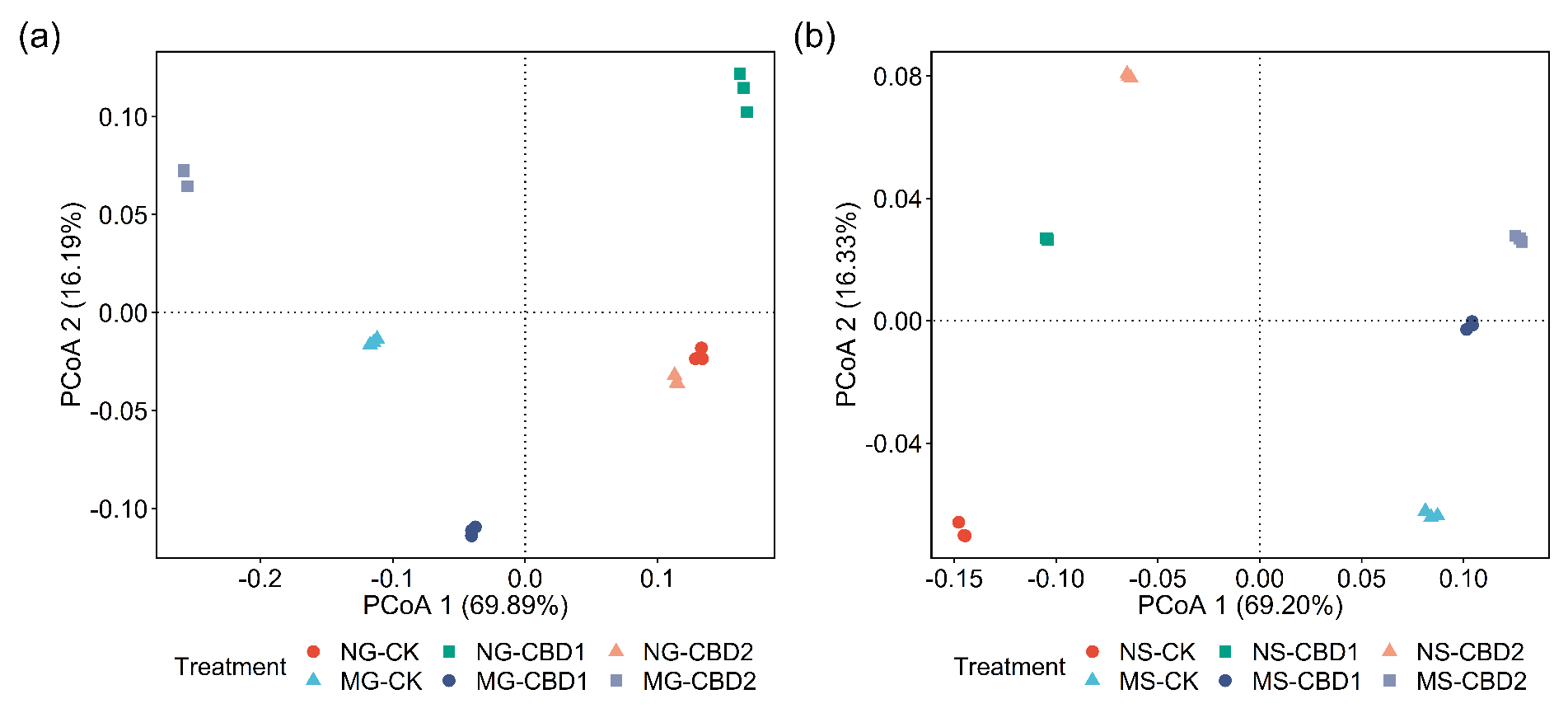


**Figure S6.** PCoA plot of bacterial communities in the earthworm gut (a) and soil (b) among treatments. NS-CK, NS-CBD1, and NS-CBD2 represent the soil samples in the un-manured soil with 0, 1.0, and 2.0 mg·kg^−1^ CBD treatment, respectively. MS-CK, MS-CBD1, and MS-CBD2 represent the soil samples in the manured soil with 0, 1.0, and 2.0 mg·kg^−1^ CBD treatment, respectively.


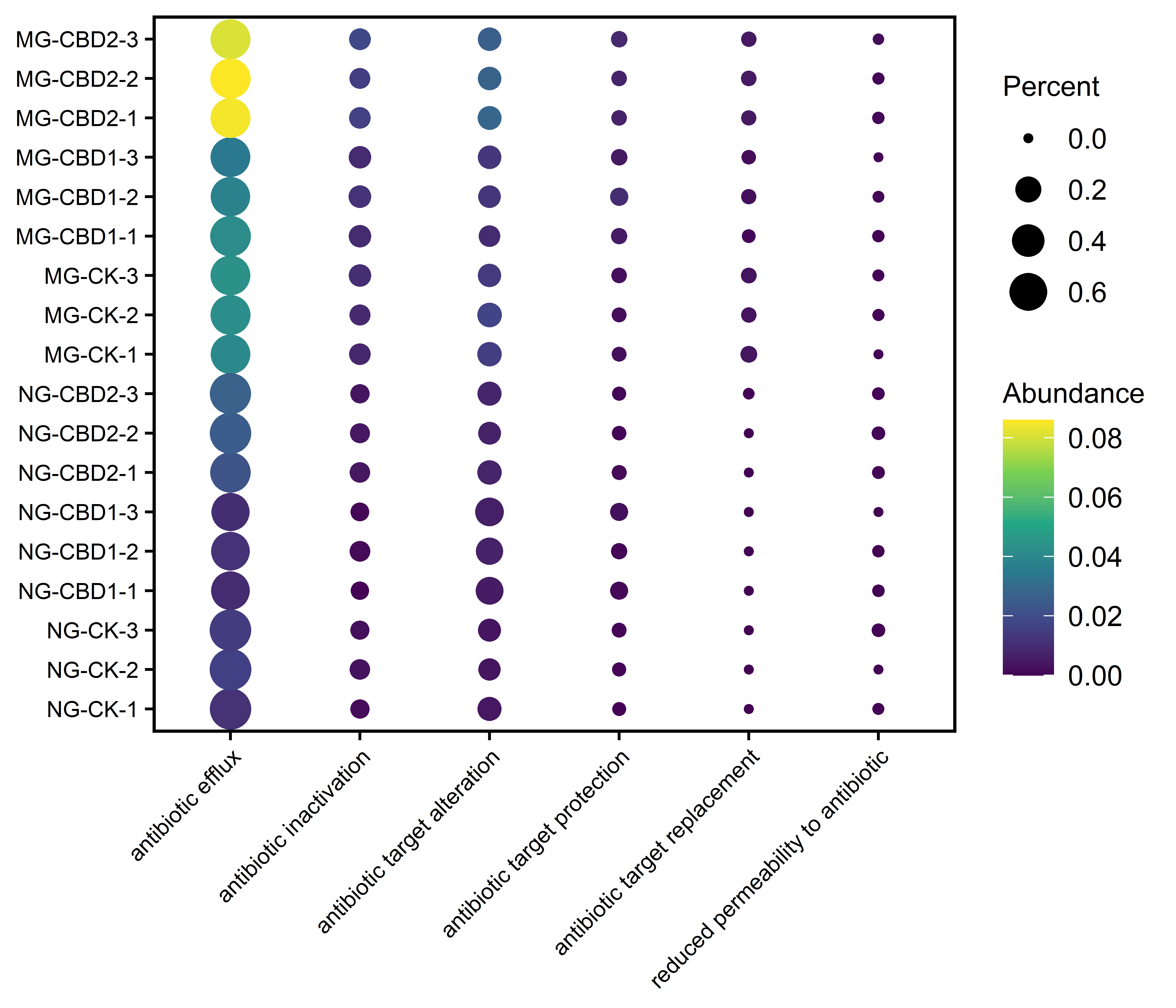


**Figure S7.** Antibiotic resistance mechanism of ARGs in the earthworm gut from the different treatments. NG-CK, NG-CBD1, and NG-CBD2 represent the earthworm gut samples in the un-manured soil with 0, 1.0, and 2.0 mg·kg^−1^ CBD treatment, respectively. MG-CK, MG-CBD1, and MG-CBD2 represent the earthworm gut samples in the manured soil with 0, 1.0, and 2.0 mg·kg^−1^ CBD treatment, respectively.


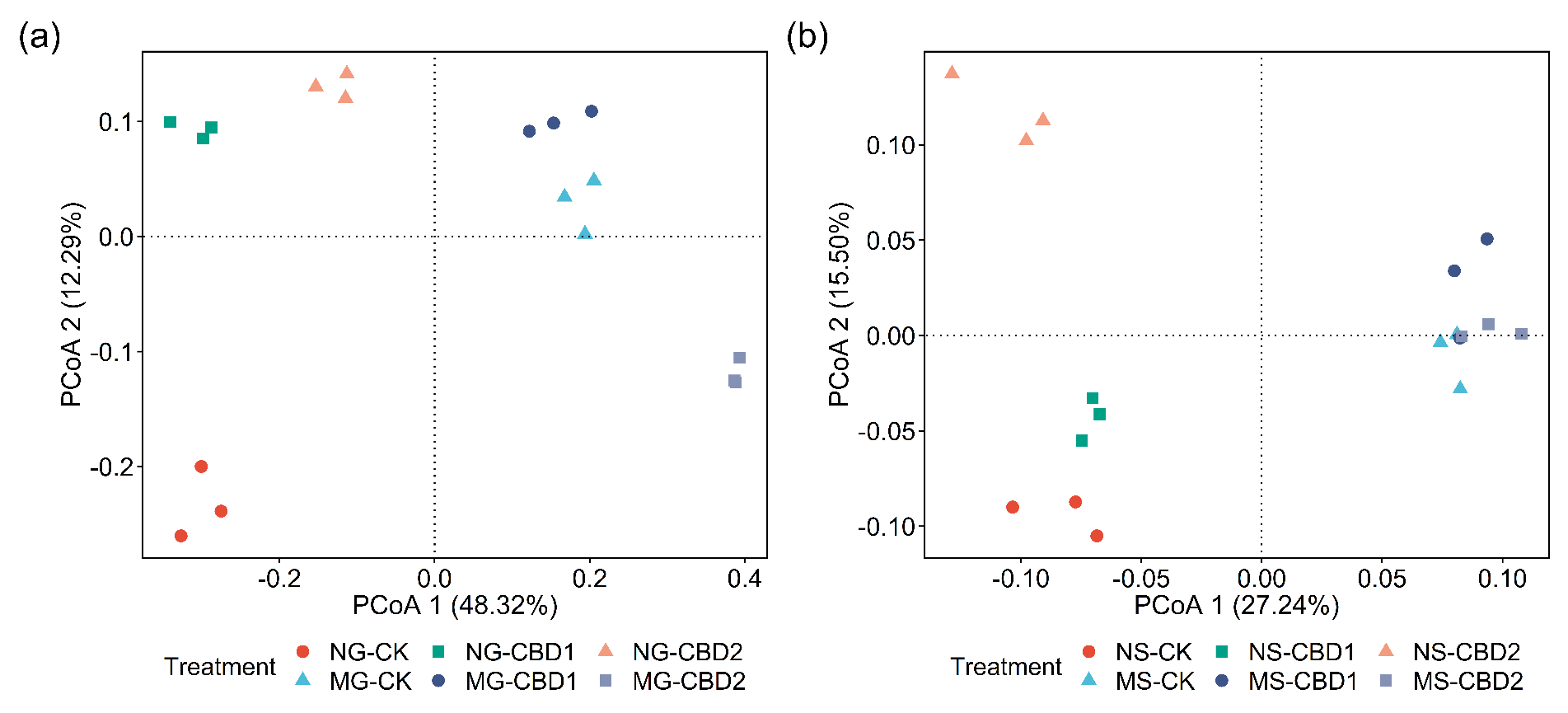


**Figure S8.** PCoA plot of ARGs profiles in the earthworm gut (a) and soil (b) among treatments. NG-CK, NG-CBD1, and NG-CBD2 represent the earthworm gut samples in the un-manured soil with 0, 1.0, and 2.0 mg·kg^−1^ CBD treatment, respectively. MG-CK, MG-CBD1, and MG-CBD2 represent the earthworm gut samples in the manured soil with 0, 1.0, and 2.0 mg·kg^−1^ CBD treatment, respectively. NS-CK, NS-CBD1, and NS-CBD2 represent the soil samples in the un-manured soil with 0, 1.0, and 2.0 mg·kg^−1^ CBD treatment, respectively. MS-CK, MS-CBD1, and MS-CBD2 represent the soil samples in the manured soil with 0, 1.0, and 2.0 mg·kg^−1^ CBD treatment, respectively.


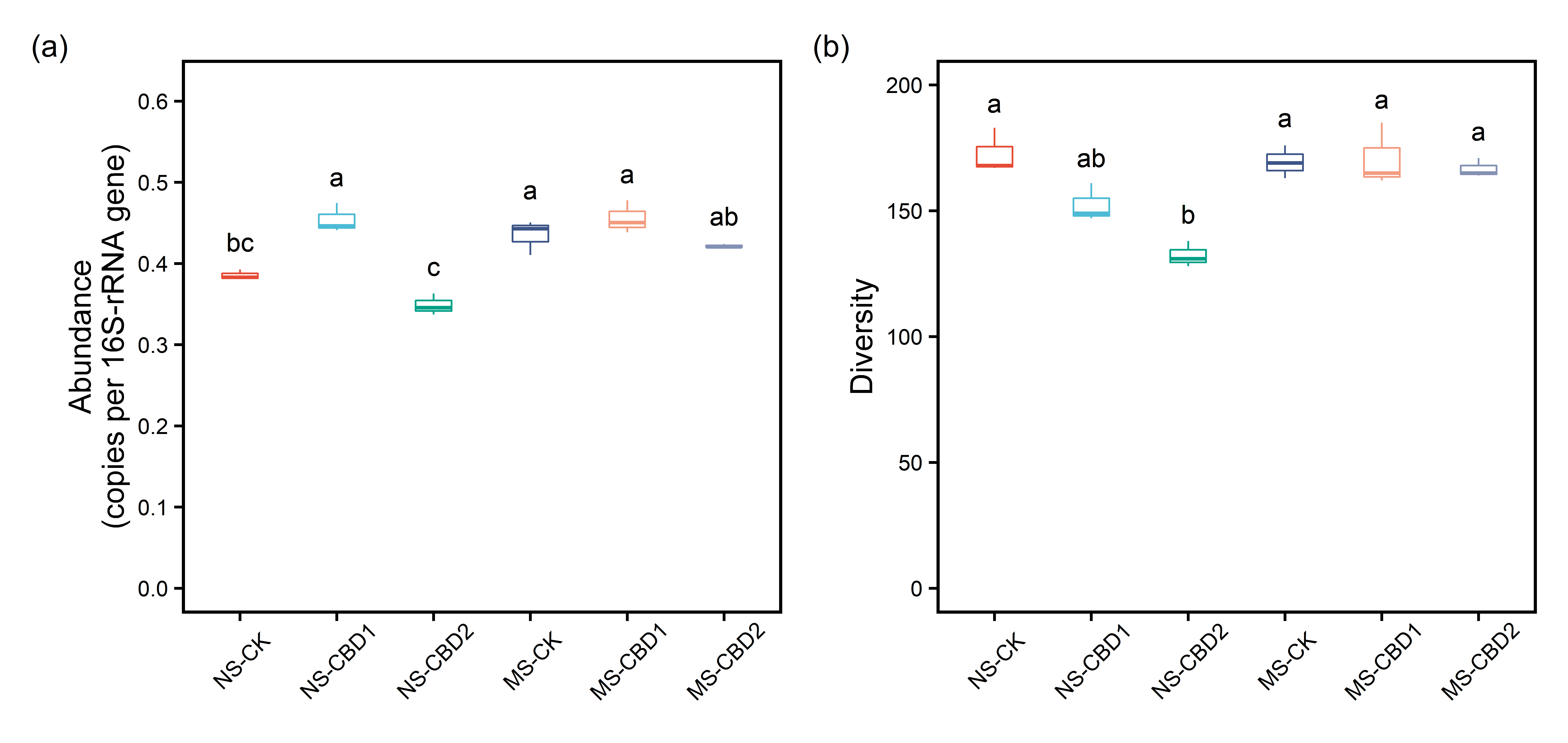


**Figure S9.** Comparison of total abundance (a) and diversity (b) of antibiotic resistance genes (ARGs) in the soil among treatments. NS-CK, NS-CBD1, and NS-CBD2 represent the soil samples in the un-manured soil with 0, 1.0, and 2.0 mg·kg^−1^ CBD, respectively. MS-CK, MS-CBD1, and MS-CBD2 represent the soil samples in the manured soil with 0, 1.0, and 2.0 mg·kg^−1^ CBD, respectively.


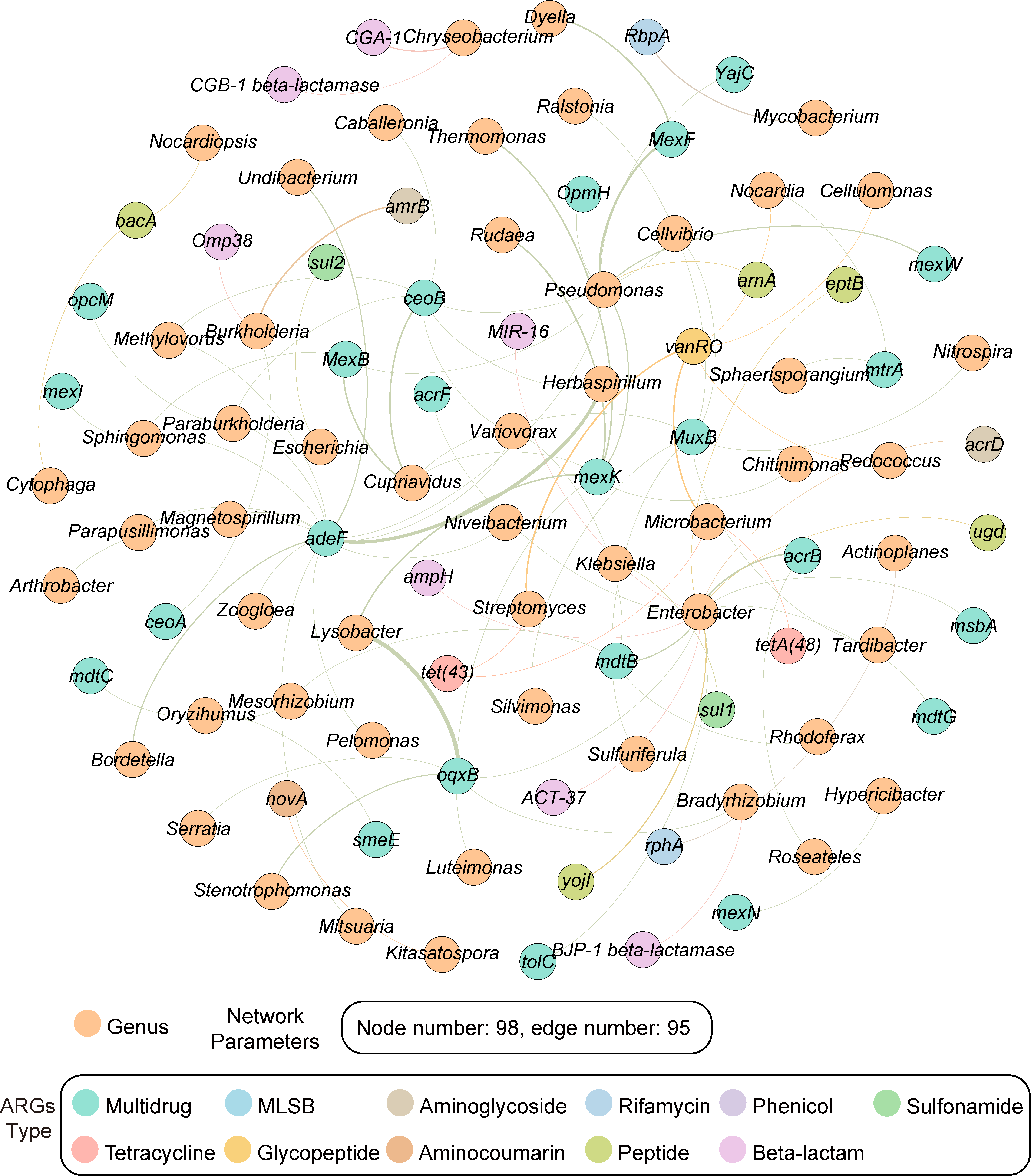


**Figure S10.** Network of ARGs hosts based on the metagenomic assembly analysis in the soil.





**Figure S11.** Pearson's correlations between ARGs and MGEs in the earthworm gut among treatments.


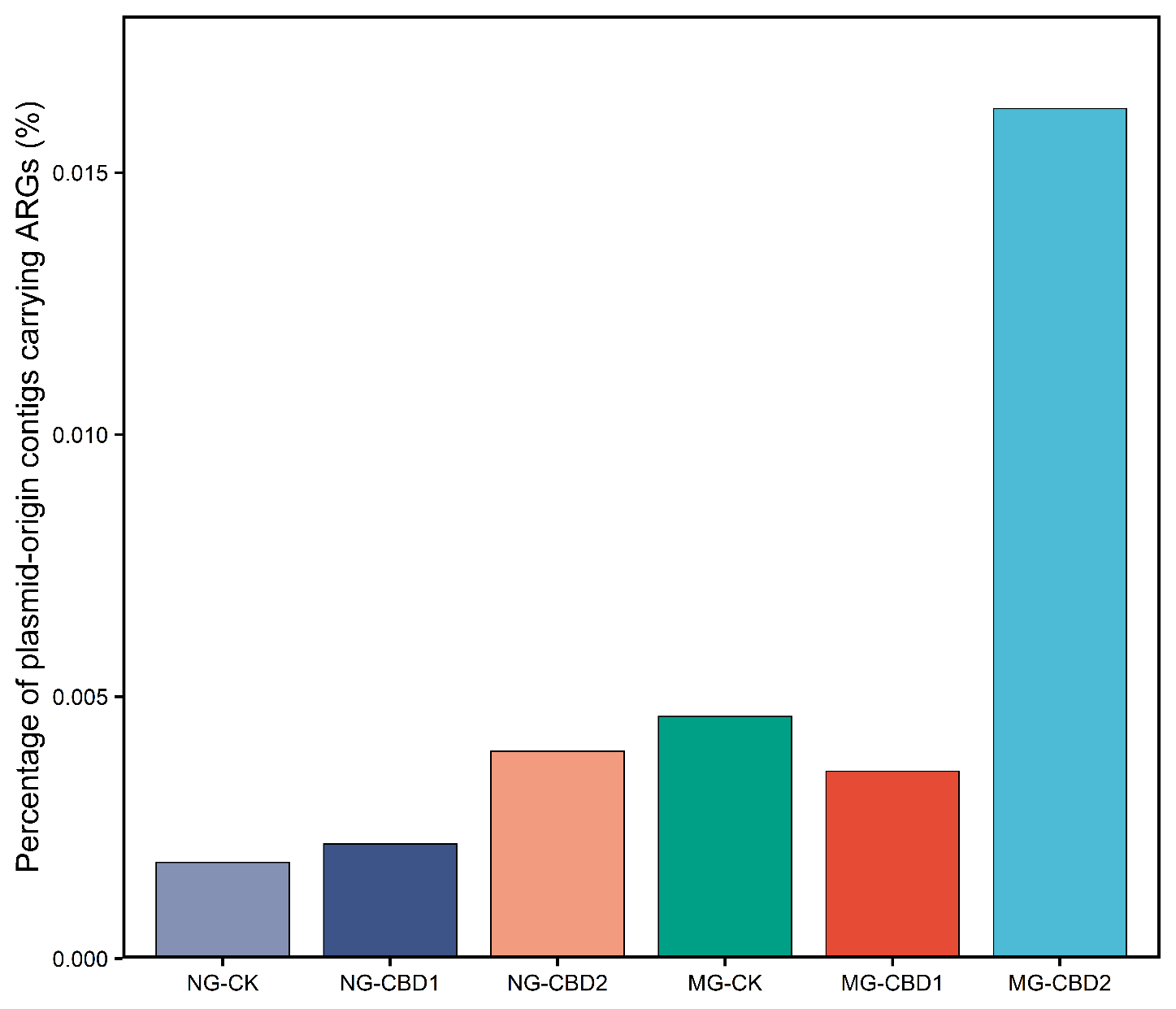


**Figure S12.** Percentage of plasmid-origin contigs carrying ARGs in the earthworm gut from the different treatments. NG-CK, NG-CBD1, and NG-CBD2 represent the earthworm gut samples in the un-manured soil with 0, 1.0, and 2.0 mg·kg^−1^ CBD, respectively. MG-CK, MG-CBD1, and MG-CBD2 represent the earthworm gut samples in the manured soil with 0, 1.0, and 2.0 mg·kg^−1^ CBD, respectively.
